# Plastid phylogenomics and fossil evidence provide new insights into the evolutionary complexity of the ‘woody clade’ in Saxifragales

**DOI:** 10.1186/s12870-024-04917-9

**Published:** 2024-04-12

**Authors:** Linbo Jia, Shuying Wang, Jinjin Hu, Ke Miao, Yongjiang Huang, Yunheng Ji

**Affiliations:** 1 CAS Key Laboratory for Plant Diversity and Biogeography of East Asia, Kunming Institute of Botany, Chinese Academy of Sciences, Kunming, 650201 China; 2 Kunming College of Life Science, University of Chinese Academy of Sciences, Kunming, 650201 China

**Keywords:** Phylogenetically recalcitrant lineage, Radiative diversification, Incomplete lineage sorting, Ancient hybridization, Ancient extinction

## Abstract

**Background:**

The “woody clade” in Saxifragales (WCS), encompassing four woody families (Altingiaceae, Cercidiphyllaceae, Daphniphyllaceae, and Hamamelidaceae), is a phylogenetically recalcitrant node in the angiosperm tree of life, as the interfamilial relationships of the WCS remain contentious. Based on a comprehensive sampling of WCS genera, this study aims to recover a robust maternal backbone phylogeny of the WCS by analyzing plastid genome (plastome) sequence data using Bayesian inference (BI), maximum likelihood (ML), and maximum parsimony (MP) methods, and to explore the possible causes of the phylogenetic recalcitrance with respect to deep relationships within the WCS, in combination with molecular and fossil evidence.

**Results:**

Although the four WCS families were identically resolved as monophyletic, the MP analysis recovered different tree topologies for the relationships among Altingiaceae, Cercidiphyllaceae, and Daphniphyllaceae from the ML and BI phylogenies. The fossil-calibrated plastome phylogeny showed that the WCS underwent a rapid divergence of crown groups in the early Cretaceous (between 104.79 and 100.23 Ma), leading to the origin of the stem lineage ancestors of Altingiaceae, Cercidiphyllaceae, Daphniphyllaceae, and Hamamelidaceae within a very short time span (∼4.56 Ma). Compared with the tree topology recovered in a previous study based on nuclear genome data, cytonuclear discordance regarding the interfamilial relationships of the WCS was detected.

**Conclusions:**

Molecular and fossil evidence imply that the early divergence of the WCS might have experienced radiative diversification of crown groups, extensive extinctions at the genus and species levels around the Cretaceous/Paleocene boundary, and ancient hybridization. Such evolutionarily complex events may introduce biases in topological estimations within the WCS due to incomplete lineage sorting, cytonuclear discordance, and long-branch attraction, potentially impacting the accurate reconstruction of deep relationships.

**Supplementary Information:**

The online version contains supplementary material available at 10.1186/s12870-024-04917-9.

## Background

The core task of plant phylogenetics is to recover the tree of life for Plantae [1–3]. Although recent molecular phylogenetic studies have greatly improved our understanding of the angiosperm tree of life, some nodes remain notoriously recalcitrant [4–8]. Notably, the taxonomic affinities of such phylogenetically recalcitrant lineages have been markedly debated among previous morphology-based classification systems [e.g., 9, 10–14]; and in recent molecular phylogenetic studies, the relationships between these nodes and related taxa were either poorly resolved or significantly incongruent [e.g., 2, 7, 15–17]. Several lines of evidence suggest that these phylogenetically recalcitrant nodes may have undergone some evolutionarily complex events, such as radiative diversification, morphological convergence, and ancient hybridization, which most likely resulted in the absence of synapomorphies and/or phylogenetic conflicts inferred from different datasets [e.g., 4, 18–20].

The “woody clade” in Saxifragales (WCS), as defined by Jian et al. [21], comprises four woody families exclusively occurring in the Northern Hemisphere, i.e., Altingiaceae (one genus, eight species) [22], Cercidiphyllaceae (one genus, two species) [23], Daphniphyllaceae (one genus, 30 species) [24, 25], and Hamamelidaceae (27 genera, 120 species) [7, 26]. The WCS represents one of the phylogenetically recalcitrant nodes in the angiosperm tree of life. The taxonomic affinities of these four families (as shown in Tables 1 and 2) are also contentious in the morphology-based classification systems proposed by Hutchinson [10], Cronquist [11], Dahlgren [12], Thorne [13], and Takhtajan [14]. This suggests a lack of robust morphological synapomorphies that unite them [21, 27]. Although previous studies have successfully recovered the phylogenetic backbone of Saxifragales and consistently resolved the WCS as a monophyletic group with strong support [8, 21, 27–33], the unresolved interfamilial relationships for Altingiaceae, Cercidiphyllaceae, Daphniphyllaceae, and Hamamelidaceae continue to be a subject of debate. Theoretically, the inconsistencies regarding the deep relationships of the WCS imply that it might have been subject to some evolutionarily complex events during its early evolution [16, 17, 19].

**Table 1:** Comparison of the order-level taxonomic placements of the four families within the “woody clade” in Saxifragales among previous morphology-based classification systems

	Classification systems				
	Hutchinson (1959)	Cronquist (1981)	Dahlgren (1983)	Thorne (1992)	Takhtajan (1997)
Altingiaceae	Hamamelidales*	Hamamelidales*	Hamamelidales*	Hamamelidales*	Hamamelidales
Cercidiphyllaceae	Magnoliales	Hamamelidales	Cercidiphyllales	Hamamelidales	Cercidiphyllales
Daphniphyllaceae	Hamamelidales	Daphiphyllales	Buxales	Buxales	Hamamelidales
Hamamelidaceae	Hamamelidales	Hamamelidales	Hamamelidales	Hamamelidales	Hamamelidales

* Altingiaceae was treated as a subfamiliar member of Hamamelidaceae by the corresponding classification system

**Table 2:** Taxonomic studies of Hamamelidaceae

Harms (1930)	Schulze-Menz (1964)	Chang (1973, 1979)	Bogle (1980)	Endress (1989, 1993)	Li (1997)
**Disanthoideae** * Disanthus*	**Disanthoideae** *Disanthus*	**Disanthoideae** *Disanthus*	**Disanthoideae** *Disanthus*	—	**Disanthoideae** *Disanthus*
**Bucklandioideae** * Bucklandia*	**Symingtonioideae** *Symingtonia*	**Exbucklandioideae** *Exbucklandia*	**Exbucklandioideae** *Exbucklandia* *Mytilaria* *Chunia*	**Exbucklandioideae** *Disanthus* *Exbucklandia* *Mytilaria* *Chunia*	**Exbucklandioideae** *Exbucklandia*
**Rhodoleioideae** * Rhodoleia*	**Rhodoleioideae** *Rhodoleia*	**Rhodoleioideae** *Rhodoleia*	**Rhodoleioideae** *Rhodoleia*	**Rhodoleioideae** *Rhodoleia*	**Rhodoleioideae** *Rhodoleia*
—	—	**Mytilarioideae** *Mytilaria* *Chunia*	—	—	**Mytilarioideae** *Mytilaria* *Chunia*
**Liquidambaroideae** * Liquidambar* * Altingia* * Mytilaria* * Ostrearia*	**Liquidambaroideae** * Liquidambar* * Altingia*	**Liquidambaroideae** *Liquidambar* *Semiliquidambar* *Altingia*	**Liquidambaroideae** *Liquidambar* *Altingia*	**Liquidambaroideae** *Liquidambar* *Semiliquidambar* *Altingia*	**Altingioideae** *Liquidambar* *Semiliquidambar* *Altingia*
**Hamamelidoideae** Hamamelideae * Hamamelis* * Loropetalum* * Tetrathyrium* * Trichocladus* * Maingaya* * Embolanthera* * Dicoryphe* Distylieae * Distylium* * Sycopsis* * Sinowilsonia* Eustigmateae * Eustigma* Corylopsideae * Corylopsis* * Fortunearia* Fothergilleae * Fothergilla* * Parrotia* * Parrotiopsis*	**Hamamelidoideae** Hamamelideae *Hamamelis* *Trichocladus* *Dicoryphe* Distylieae *Distylium* *Sycopsis* Eustigmateae *Eustigma* Corylopsideae *Corylopsis* *Fortunearia* *Sinowilsonia* Fothergilleae *Fothergilla* *Parrotia* *Parrotiopsis*	**Hamamelidoideae** Hamamelideae *Hamamelis* *Loropetalum* *Tetrathyrium* Distylieae *Distylium* *Sycopsis* *Sinowilsonia* Eustigmateae *Eustigma* Corylopsideae *Corylopsis* *Fortunearia*	**Hamamelidoideae** Hamamelideae *Hamamelis* *Loropetalum* *Tetrathyrium* *Trichocladus* *Maingaya* *Embolanthera* *Dicoryphe* *Ostrearia* *Neostrearia* Distylieae *Distylium* *Sycopsis* *Distyliopsis* *Molinadendron* *Matudaea* Eustigmateae *Eustigma* Corylopsideae *Corylopsis* *Fortunearia* *Sinowilsonia* Fothergilleae *Fothergilla* *Parrotia* *Parrotiopsis*	**Hamamelidoideae** Hamamelideae 1. Hamamelidineae *Hamamelis* 2. Loropetalineae *Loropetalum* *Tetrathyrium* *Maingaya* *Embolanthea* 3. Dicoryphineae *Dicoryphe* *Trichocladus* *Ostrearia* *Neostrearia* *Neohdendron* Eustigmateae *Eustigma* *Fortunearia* *Sinowilsonia* Corylopsideae *Corylopsis* Fothergilleae *Molinadendron* *Fothergilla* *Parrotia* *Parrotiopsis* *Sycopsis* *Distyliopsis* *Distylium* *Matudaea*	**Hamamelidoideae** Hamamelideae *Hamamelis* Loropetaleae *Loropetalum* *Tetrathyrium* *Maingaya* *Embolanthea* *Matudaea* Dicorypheae *Dicoryphe* *Trichocladus* *Ostrearai* *Neostrearia* *Neohdendron* Eustigmateae *Eustigma* *Fortunearia* *Sinowilsonia* *Molinadendron* Corylopsideae *Corylopsis* Fothergilleae *Fothergilla* *Parrotia* *Shaniodendron* *Parrotiopsis* *Sycopsis* *Distyliopsis* *Distylium*

Given that fossils represent the remains of past plants, they have the capacity to unveil intermediate evolutionary connections from the geological past, thereby offering valuable evidence for comprehending the evolution of the tree of life [34–37]. The combination of molecular and fossil evidence is recommended as an efficient approach for inferring the evolutionarily complex events that might result in phylogenetic recalcitrance in the angiosperm tree of life [16, 30, 38, 39]. Recently, genome-scale sequence data, particularly plastid genomes (plastomes), have been increasingly used to solve historical puzzles in plant phylogenetics [e.g, 8, 28, 29, 40–48]. In general, phylogeny inferred from uniparentally inherited plastomes reflects only the maternal (or, in some cases, paternal) relationships, as compared to the more comprehensive evolutionary history recovered through the phylogenetic analyses of biparentally inherited nuclear genomes [49]. Nevertheless, the comparison of tree topologies inferred from plastomes and nuclear genomes can facilitate the determination of phylogenetic discordance between plastid and nuclear datasets (cytonuclear discordance), providing robust evidence to infer whether the evolution of the taxa in question has involved evolutionarily complex events, such as incomplete lineage sorting and hybridization [50, 51].

Recent phylogenomic studies have yielded valuable insights into the deep relationships within the WCS. Based on nuclear genome data generated by target-capture sequencing and RNA-seq, Folk et al. [28] recovered the phylogenetic backbone of the WCS with high-resolution and well-supported interfamilial relationships. In contrast, previous plastome-based phylogenetic analyses of the WCS taxa have demonstrated tree topologies with low support [52, 53] and inconsistent inter-familial relationships [32, 33]. Consequently, the family-level maternal relationships within the WCS remain ambiguous, providing weak evidence for detecting cytonuclear discordance in the deep relationships within the WCS. Notably, the maternal inheritance of plastomes in the WCS as well as in the Saxifragales has been verified by the investigations of several species including *Hamamelis virginiana, Heuchera micrantha*, and *Tolmiea menziesii* [54, 55]. By expanding the taxonomic sampling of the WCS to include the majority of genera, the objectives of this study are to recover a robust maternal backbone phylogeny of the WCS through analysis of plastome sequence data. Based on a time-calibrated phylogenetic framework, fossil evidence, and a comparison of plastome (this study) and nuclear [28] phylogenies, evolutionarily complex events putatively responsible for the phylogenetic recalcitrance of the deep phylogeny of the WCS were inferred.

## Results

### Plastome features

The sampled WCS plastomes showed a typical quadripartite structure, encompassing a large single-copy (LSC) region and a small single-copy (SSC) region separated by two inverted repeat (IRa and IRb) regions (Fig. S1; Tables S1, S2 and S3). The genome sizes ranged from 158,149 bp to 160,861 bp, and GC content varied from 37.7 to 38.2% (Table S3). The WCS plastomes were highly conserved in terms of gene content and structure (Fig. S2; Tables S3 and S4), and they possessed 115 unique genes, including 81 protein-coding genes (PCGs), 30 transfer RNAs (tRNAs), and four ribosomal RNAs (rRNAs). Except for the pseudogenization of *ycf15* in *Chunia bucklandioides*, none gene deletion was found in the WCS plastomes (Fig. S2).

### Phylogenetic relationship

**Fig. 1:**
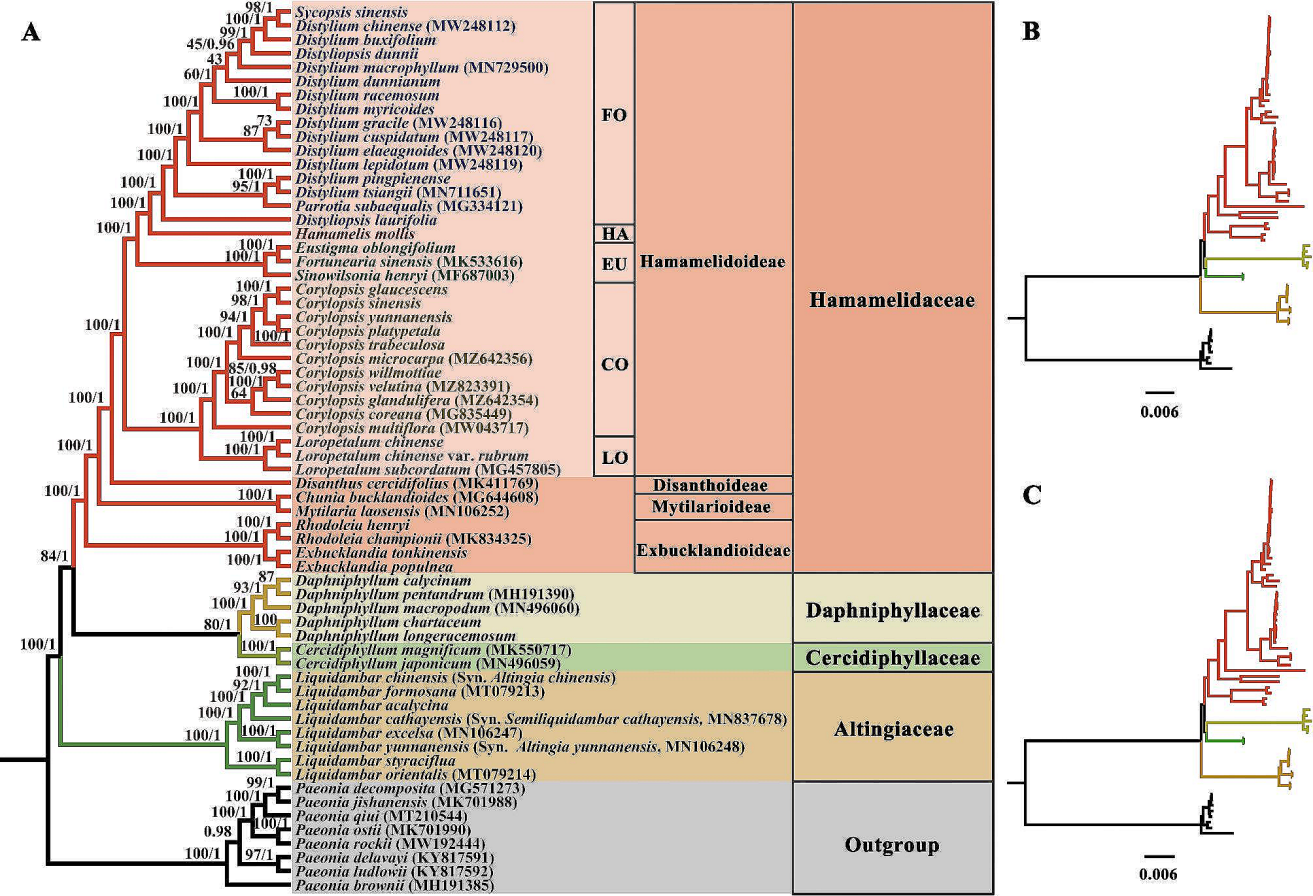
Phylogenetic relationships within the “woody clade” in Saxifragales. The tree was built using maximum likelihood (ML) and Bayesian inference (BI) methods, based on 78 plastid protein-coding genes from 64 different species. (**A**) Cladogram. (**B**) Phylogram based on ML. (**C**) Phylogram based on BI. Numbers superimposed on the branches indicate bootstrap support (%) and posterior probability. FO, Fothergilleae; HA, Hamamelideae; EU, Eustigmateae; CO, Corylopsideae; LO, Loropetaleae

**Fig. 2:**
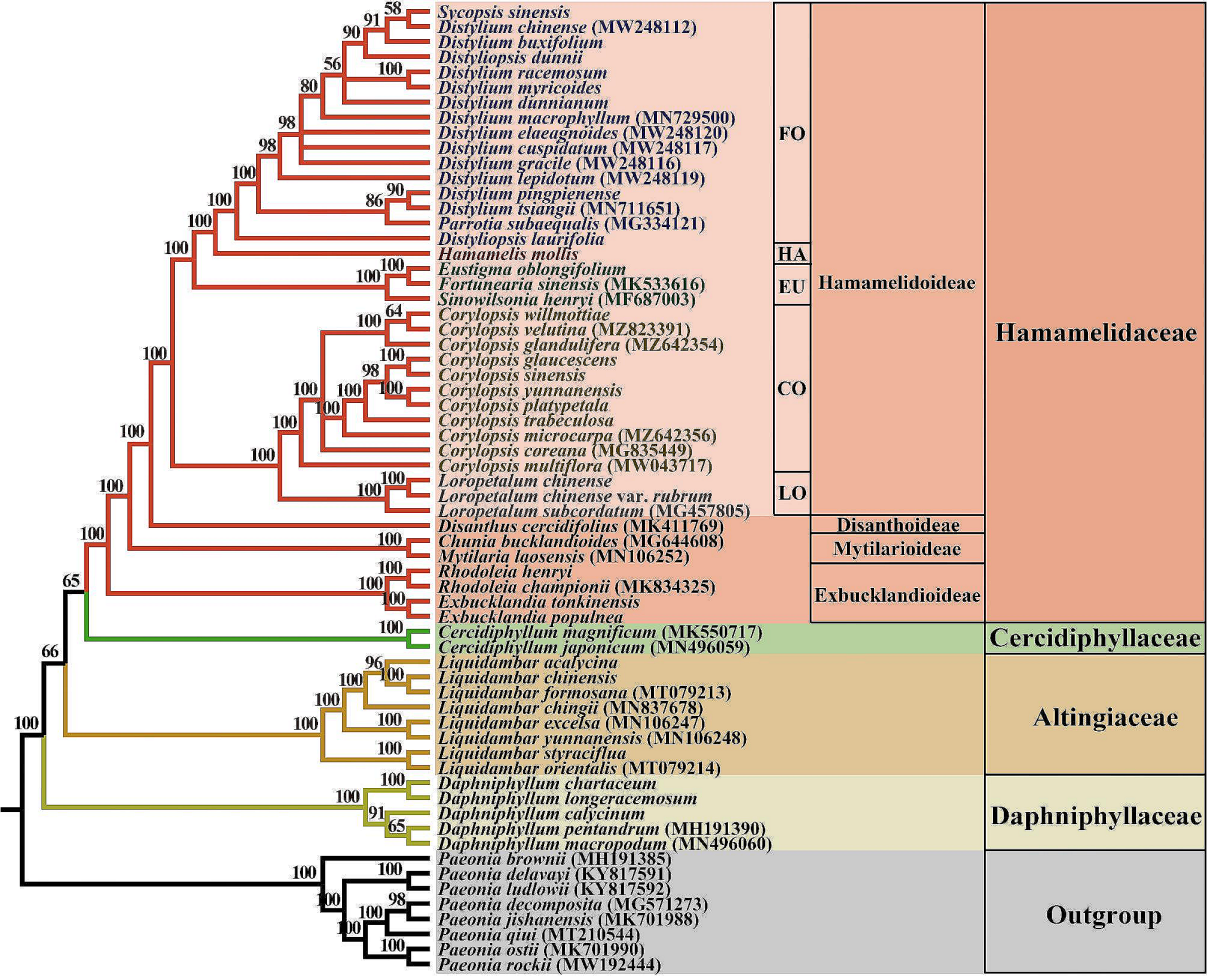
Phylogenetic relationship within the “woody clade” in Saxifragales. The tree was constructed using maximum parsimony (MP) methods, based on 78 plastid protein-coding genes of 64 species. Numbers superimposed on the branches indicate bootstrap support (%). FO, Fothergilleae; HA, Hamamelideae; EU, Eustigmateae; CO, Corylopsideae; LO, Loropetaleae

ML and BI analyses yielded highly congruent tree topologies (Figs. S3 and S4), which resolved Altingiaceae, Cercidiphyllaceae, Daphniphyllaceae, and Hamamelidaceae as fully supported [posterior probability (PP) = 1.0, bootstrap support (MLBS) = 100%] monophyletic lineages (Fig. 1). Within the WCS, the successive sister relationship of Altingiaceae (PP = 1.0, MLBS = 100%), the clade including Cercidiphyllaceae and Daphniphyllaceae (PP = 1.0, MLBS = 80%), and Hamamelidaceae (PP = 1.0, MLBS = 84%), were recovered. The MP reconstruction also fully supported the monophyly of Altingiaceae, Cercidiphyllaceae, Daphniphyllaceae, and Hamamelidaceae. However, the interfamilial relationships (the successive sister relationships of Daphniphyllaceae, Altingiaceae, Cercidiphyllaceae, and Hamamelidaceae) that recovered by the MP phylogeny is different from those resolved by the BI and ML phylogenies (Fig. 2).

### Estimation of divergence time

**Fig. 3:**
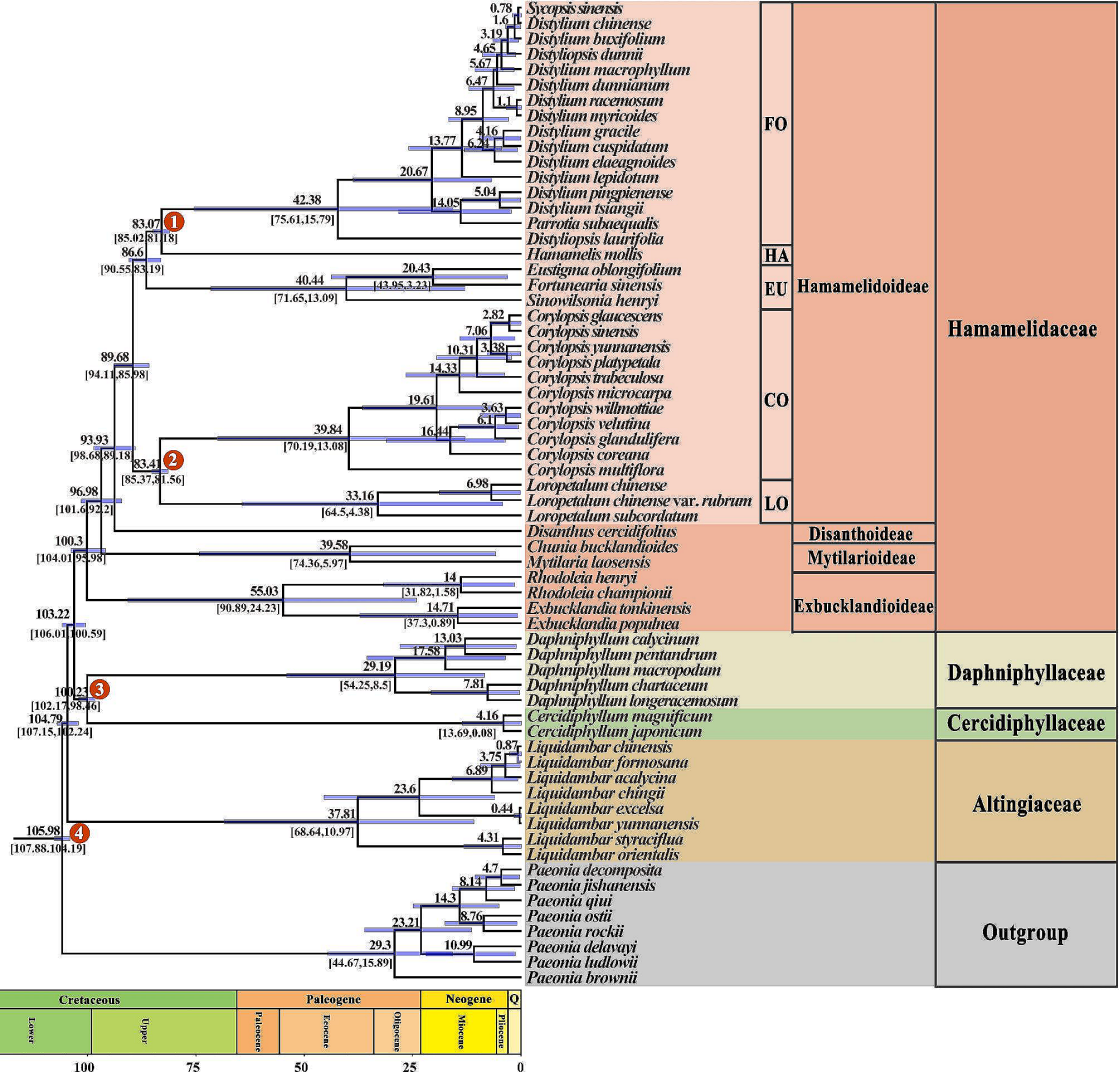
Fossil-calibrated molecular phylogenetic dating of the “woody clade” in Saxifragales. The chronological estimates were performed using MCMCtree with the maximum likelihood (ML) tree as a topological constraint. Numbers superimposed on the branches represent mean divergent ages and 95% confidence interval of each node, respectively. The red dots represent the secondary calibration and fossil-calibration nodes. The divergence time and timeline are indicated in million years ago (Ma). FO, Fothergilleae; HA, Hamamelideae; EU, Eustigmateae; CO, Corylopsideae; LO, Loropetaleae

Fossil-calibrated molecular dating (Fig. 3) showed that the diversification of the WCS crown groups initiated at ∼104.79 Ma, corresponding to the divergence of Altingiaceae. Subsequently, the divergence of Hamamelidaceae and the stem lineage ancestor of Cercidiphyllaceae and Daphniphyllaceae occurred at ∼103.22 Ma, followed by the separation of Cercidiphyllaceae and Daphniphyllaceae at ∼100.23 Ma. Despite the ancient origins of Altingiaceae, Daphniphyllaceae, and Cercidiphyllaceae during the early Cretaceous, their crown ages were dated at ∼37.8 Ma (Altingiaceae), ∼29.19 Ma (Daphniphyllaceae), and ∼4.16 Ma (Cercidiphyllaceae), respectively. Within Hamamelidaceae, the estimated ages for the origins of the subfamilies Exbucklandiodeae and Mytilarioideae were approximately 100.3 Ma and 96.98 Ma, respectively; in contrast, their crown ages were dated at around 55.03 Ma and 39.58 Ma. Additionally, the divergence between subfamilies Disanthoideae and Hamamelidoideae was estimated to have occurred approximately 93.93 (Ma), and the divergence of the five tribes within subfamily Hamamelidoideae was dated at around 86.6 Ma for tribe Eustigmateae, approximately 83.41 Ma between tribes Corylopsideae and Loropetaleae, and about 83.07 Ma between tribes Fothergilleae and Hamamelideae; by comparison, the crown ages of the tribes Fothergilleae, Eustigmateae, Corylopsideae, and Loropetalea were estimated to be around 42.38 Ma, 40.44 Ma, 39.84 Ma, and 33.16 Ma, respectively.

## Discussion

The fossil-calibrated plastome phylogeny (Fig. 3) showed that all three of the WCS monotypic families (i.e., Altingiaceae, Cercidiphyllaceae, and Daphniphyllaceae) are deep clades, considering the ancient origin of their stem lineage ancestors (104.79–100.23 Ma) versus the relatively hysteretic divergence of their crown groups (37.81–4.61 Ma). Within Hamamelidaceae, a similar pattern of early stem ages (100.3-83.07 Ma) compared to relatively later crown ages (55.03–33.16 Ma) was observed in the subfamilies Exbucklandioideae, Mytilarioideae, and Disanthoideae, as well as in the five tribes (Corylopsideae, Eustigmateae, Fothergilleae, Hamamelideae, and Loropetaleae) belonging to the subfamily Hamamelidoideae. As suggested in previous studies [8, 16, 21, 27, 29], the presence of deep stems within these clades may be attributed to a lack of cladogenesis or extensive extinction of closely related taxa during their early evolutionary processes.

Fossil evidence has disproven the hypothesis that cladogenesis was absent during the early evolution of the aforementioned WCS clades. The most typical example is Altingiaceae, within which three extinct genera, i.e., *Paleoaltingia*, *Protoaltingia*, and *Microaltingia*, have been documented from the late Cretaceous of North America [56–58]. Similarly, three extinct genera (*Allonia*, *Androdecidua*, and *Archamamelis*) have been discovered in Hamamelidaceae [59–61]. Among them, *Allonia* and *Androdecidua* were delineated based on floral remains from the late Cretaceous of the United States and were assigned to tribe Loropetaleae of subfamily Hamamelidoideae [60, 62]. *Archamamelis* was also established based on floral remains with in situ pollen from the late Cretaceous of Sweden and was assigned to tribe Hamamelideae of subfamily Hamamelidoideae [59, 62]. Within Cercidiphyllaceae, four extinct genera (*Jenkinsella*, *Joffrea*, *Nyssidium*, and *Trochodendroides*) have been reported [63–66]. Among them, a *Joffrea* species has been documented from the late Paleocene of Canada [64]; 10 *Jenkinsella*, 13 *Nyssidium*, and 60 *Trochodendroides* species [67, 68] have been discovered across the Northern Hemisphere spanning from early Cretaceous to Eocene epochs [67, 68]. Most of these extinct taxa have been identified through well-preserved reproductive organs utilizing various approaches such as scanning electron microscopy and cladistic analysis to explore their taxonomic affinities. The aforementioned evidence strongly supports that cladogenesis did occur during the early evolution of WCS clades. Although no Daphniphyllaceae fossil has yet been found, the currently available fossil evidence robustly supports the conclusion that the deep stems of the afore-mentioned WCS clades can be attributed to the prominent extinction of their closely related taxa during their initial evolutionary stages.

Notably, the majority of extinct genera within the WCS (i.e., *Allonia*, *Androdecidua*, *Archamamelis*, *Paleoaltingia*, *Protoaltingia*, and *Microaltingia*) disappeared from the fossil record after the Cretaceous period [56, 57, 59, 60, 62, 69]. Although the survival of *Jenkinsella*, *Nyssidium*, and *Trochodendroides* (Cercidiphyllaceae) extended to the Eocene, their species diversity had significantly declined since the Cretaceous/Paleocene boundary [65] and completely vanished prior to the Oligocene era [63–65, 67]. The fossil evidence suggests the extinction events in the early evolution of the WCS might have primarily occurred around the Cretaceous/Paleocene boundary. This temporal coincidence aligns with Earth’s most recent mass extinction event when over 75% of species became extinct [70–72].

For phylogenetic analysis, the families Altingiaceae, Cercidiphyllaceae, and Daphniphyllaceae exhibit extremely deep stems that may expose them to long-branch attraction [73], potentially leading to erroneous grouping in evolutionary trees. Previous studies have demonstrated that MP phylogenies are more susceptible to long-branch attraction compared to BI and ML phylogenies [74, 75]. Consistent with this, our MP phylogeny revealed distinct tree topologies concerning the interfamilial relationships among Altingiaceae, Cercidiphyllaceae, and Daphniphyllaceae when compared to ML and BI phylogenies (Figs. 1 and 2). These findings suggest that the reconstruction of the WCS’s phylogeny could be influenced by long-branch attraction effects, resulting in a certain degree of bias in topological estimation [73, 75, 76]. Consequently, significant extinction events during the early evolution of the WCS might contribute significantly to the recalcitrance observed in resolving deep relationships within this clade.

Previous studies have revealed that both BI and ML phylogenies are more resistant to long-branch attraction than MP phylogenies [73–75]. The following discussion is mainly based on the BI and ML tree topologies because the interfamilial relationships recovered from the BI and ML phylogenies can be more reliable than those recovered from the MP phylogenies. In this study, both BI and ML phylogenies recovered three successively divergent clades within the WCS, corresponding to Altingiaceae, Cercidiphyllaceae + Daphniphyllaceae, and Hamamelidaceae. The interfamilial relationships are consistent with those revealed by Jian et al. [21], Soltis et al. [27], Xiang et al. [30], Tarullo et al. [31], and Bi et al. [33]. Nevertheless, the interfamilial relationships recovered in this study are inconsistent with those obtained by analyzing plastid sequence data alone, which proposed topologies of (Daphniphyllaceae, (Altingiaceae, (Hamamelidaceae, Cercidiphyllaceae))) [32, 77], (Altingiaceae, (Cercidiphyllaceae, (Daphniphyllaceae, Hamamelidaceae))) based on 83 plastid PCGs of four species [52], (Hamamelidaceae, (Cercidiphyllaceae, Daphniphyllaceae, Altingiaceae)) based on 83 protein-coding genes, and ((Daphniphyllaceae, Altingiaceae), (Hamamelidaceae, Cercidiphyllaceae)) based on plastome of nine species [53]. Due to the limited taxonomic sampling of the WCS, these studies likely suffered from phylogenetic errors. Based on a more comprehensive sampling of the WCS taxa at the genus level and the concatenated 78 plastid PCGs that contains more sequence variations and phylogenetically informative sites than was available in previous plastid phylogenetic studies, the deep relationships within WCS recovered in this study were robustly supported. This led to the recovery of the maternal backbone phylogeny of the WCS, providing new insights for inferring the evolutionarily complex events that likely caused the phylogenetic recalcitrance of the deep relationships within WCS.

**Fig. 4:**
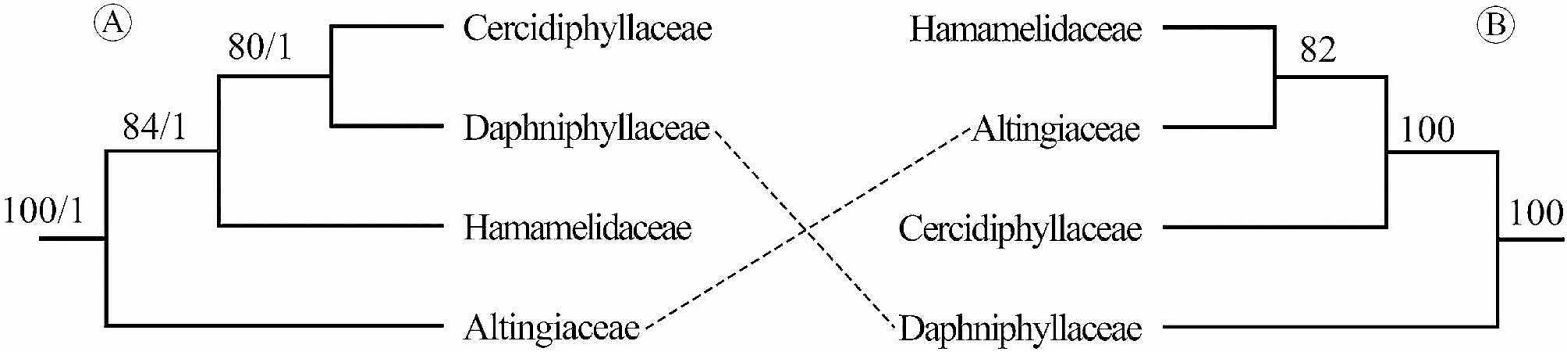
Comparison of deep relationships within the “woody clade” in Saxifragales. (**A**) Phylogenetic analyses of plastomes. (**B**) Phylogenetic analyses of target-capture sequencing and transcriptome data

Topologically, the deep relationships of the WCS recovered in this study are incongruent with those recovered based on the phylogenomic analysis of target-capture sequencing and transcriptome data [28], which proposed a successive divergence of Daphniphyllaceae, Cercidiphyllaceae, Altingiaceae, and Hamamelidaceae with robust support for each node. Based on these results, phylogenetic incongruence between plastid and nuclear data (cytonuclear discordance) was detected in the deep clades of the WCS (Fig. 4). Cytonuclear discordance is commonly observed in some phylogenetically recalcitrant plant lineages [16, 50, 78–81]; in most cases, nuclear phylogeny is more congruent with morphologic characteristics than plastid phylogeny, and such discordance is thought to be caused by hybridization [17, 50, 82, 83].

By comparing the plastome (this study) and nuclear genome phylogenies [28] with the morphological characteristics, we found that the interfamilial relationships recovered based on the analyses of target-capture sequencing and transcriptome data [28] are more consistent with the morphologies. Specifically, Altingiaceae has traditionally been treated as a member of Hamamelidaceae [10, 11, 13]. Both families are monoecious and have one or two-chambered anthers, distinct from the dioecism and four-chambered anthers of Cercidiphyllaceae and Daphniphyllaceae [23, 24, 26, 84]. These morphological affinities justify the close relationship between Altingiaceae and Hamamelidaceae. In addition, Altingiaceae, Cercidiphyllaceae, and Hamamelidaceae have stipules and winged seeds, distinguishing them from the non-stipulate leaves and wingless seeds of Daphniphyllaceae [23, 24, 26, 84], supporting the transitional position of Cercidiphyllaceae as a phylogenetic link between Daphniphyllaceae and the clade Altingiaceae + Hamamelidaceae. The high levels of consistency between the nuclear genome phylogeny and morphological traits suggest that the cytonuclear discordance observed in the WCS phylogeny might have been caused by ancient interfamilial hybridization.

Interestingly, a previous study [56] showed that interfamilial hybridization might have occurred during the early evolution of the WCS. Based on scanning electron microscopic investigations, tricolpate-reticulate pollen was found to attach to the stigmas of the fossil pistillate inflorescences of *Microaltingia*, an extinct Altingiaceae genus from the late Cretaceous of the United States [56, 85]. Within the WCS, tricolpate-reticulate pollen exists in Hamamelidaceae, Cercidiphyllaceae, and Daphniphyllaceae but not in Altingiaceae [56, 85]. The fossil evidence implies that ancient hybridization between Altingiaceae and closely related families might be feasible. Therefore, chloroplast capture [17, 82] caused by ancient hybridization is likely a reasonable interpretation of the phylogenetic discordances detected in the deep relationships within the WCS.

Concordant with fossil evidence [56, 57, 59, 60, 62, 69, 86], which suggests that the early evolution of the WCS might have experienced radiative lineage diversification, the results of divergence time estimation indicated that the WCS underwent rapid divergence in the crown groups during the early Cretaceous, leading to the occurrence of the stem lineage ancestors of Altingiaceae, Cercidiphyllaceae, Daphniphyllaceae, and Hamamelidaceae within a very short time span (within ∼4.56 Ma, and between 104.79 and 100.23 Ma). The mutually supporting evidence suggests that, in addition to ancient hybridization, incomplete lineage sorting (the stochastic sorting of ancestral sequence polymorphisms) [16, 51] resulted from the process of radiative diversification during the early evolution of WSC may contribute to the observed phylogenetic recalcitrance in the deep relationships of the WCS.

## Conclusion

The WCS is a representative of the phylogenetically recalcitrant node in the angiosperm tree of life, within which the deep relationships remain poorly resolved. Based on a broad taxonomic sampling at the genus level across the four currently recognized families in the WCS, we recovered a robust maternal backbone phylogeny for this group. Based on molecular and fossil evidence, this study indicates that the early evolution of the WCS might have undergone radiative diversification of crown groups in the early Cretaceous, ancient hybridization, and prominent extinction events during the transition between the Cretaceous and Paleocene. These events most likely resulted in the phylogenetic reconstruction of the deep relationships within the WCS being adversely affected by incomplete lineage sorting, cytonuclear discordance, and long-branch abstraction, which inevitably led to serious topological estimation biases. Nevertheless, possible ancient hybridization and incomplete lineage sorting in the early evolution of the WCS inferred in this study are merely based on the phylogenetic incongruence between plastid and nuclear genomic data, and other supporting evidence is still lacking. This inference needs to be validated through in-depth analysis of the conflicts between the species tree and gene trees using nuclear genome data containing multiple single-copy orthologous genes.

## Methods

### Taxonomic sampling, DNA extraction, and Illumina sequencing

According to the most recent taxonomic revision of Altingiaceae [22], Cercidiphyllaceae [23], Daphniphyllaceae [24, 25], and Hamamelidaceae [26], the WCS comprises approximately 173 species belonging to 30 genera [7, 22, 23, 25]. In this study, 56 species representing all three genera in the three monotypic families (Cercidiphyllaceae, Daphniphyllaceae, and Altingiaceae) and 15 out of the 27 currently recognized genera in Hamamelidaceae were sampled. Among these, the plastomes of 27 species representing 10 genera were newly sequenced in this study (Table S1), and publicly available plastomes of 29 species representing 13 genera across four WCS families were obtained from the NCBI GenBank database (Table S2).

The genomic DNA of these newly sequenced samples was extracted from approximately 100 mg of silica gel-dried leaf tissue using a modified CTAB method [87]. Then, the shotgun DNA libraries were constructed using a TruSeq DNA Sample Prep Kit (Illumina, Inc., San Diego, CA, USA) following the manufacturer’s instructions. Paired-end sequencing was performed on an Illumina NovaSeq 6000 platform to generate approximately 4 Gbp of raw reads for each sample.

### Plastome assembly and annotation

Trimmomatic v0.40 [88] was used to filter low-quality Illumina raw reads with pre-set parameters. The pipeline GetOrganelle v1.7.7.0 [89] was employed to assemble plastomes using Illumina clean reads with default parameters, using the plastomes of the corresponding species as a reference (Table S2). The assembled plastomes were further adjusted using Bandage v8.0 [90] and annotated using the online program GeSeq [91]. Information on the initiation codon, stop codon, and intron sites of the protein-coding genes was examined and manually adjusted using Geneious v10.2 [92]. tRNA genes were annotated using trnascan-SE v2.0 [93]. The inverted repeat (IRa and IRb) regions of the plastome were determined using Geneious v10.2 [92]. All WCS plastomes were progressively aligned with the complete plastome of *Hamamelis mollis*, as a reference, using the multiple genome alignment tool Mauve v4.0 [94], after one of the inverted repeat regions was removed.

### Phylogenetic analysis

Given the close relationship between the WCS and Paeoniaceae [e.g., 27, 52], eight *Paeonia* species were selected as outgroups. In total, 78 plastid PCGs (Table S4) commonly shared by the sampled plastomes were extracted from each plastome for phylogenetic analysis using PhyloSuite v1.2.2 [95]. The PCGs were aligned with MAFFT v7.402 [96] and concatenated using Geneious v10.2 [92], with the default parameters.

BI, ML, and MP methods were used to infer phylogenetic relationships. For BI analysis, PartitionFinder v2.1.1 [97] was used to estimate the best partitioning schemes and substitution models (Table S5). MrBayes v3.22 [98] based on BI, was used to construct a phylogenetic tree. The Markov Chain Monte Carlo (MCMC) analyses were run for two million generations, sampling one tree every 100 generations and discarding the first 25% of trees as burn-in. The obtained trees were evaluated for convergence using Tracer v1.7.1 [99], with effective sample sizes (ESSs) > 200. For the ML analysis, IQ-tree v2.1.3 [100, 101] was used to estimate the best partitioning schemes and substitution models (Table S6), and the ML bootstrap support (MLBS) value of each branch was calculated with 1000 replicates. The MP analyses employed PAUP* 4.a168 on the XSEDE platform via the Cyberinfrastructure for Phylogenetic Research Science (CIPRES) Gateway web server. All characters were treated as unordered and equally weighted, while branches with a minimum optimized length of zero were condensed [102]. The analysis utilized a heuristic search approach with tree bisection-reconnection (TBR) branch swapping. It generated 1000 replicates, employing random-addition-sequence methodology and allowing for the storage of up to 10,000 trees per replicate. From the retained most-parsimonious trees (MPTs), a strict consensus tree was derived. Bootstrap support values were calculated using bootstrap analyses [103] involving 1000 replicates. Each replicate consisted of 10 random-addition-sequence replicates, with a maximum of 100 trees saved per replicate.

### Fossil record and estimation of divergence time

Data for the fossil record of the WCS were obtained from the literature, the Paleobiology Database (http://paleobiodb.org/), and the International Fossil Plant Names Index (http://www.ifpni.org/). BEAST v1.10.4 [104] was employed to estimate the divergence time. According to the most recent time-calibrated angiosperm phylogeny [29], the stem age of the WCS is constrained to 105.2 Ma. In addition, the earliest fossils of the extinct genera *Trochodendroides* [66], *Allonia*/*Androdecidua* [60, 62], and *Archamamelis* [59, 61, 62] were chosen to constrain the stem ages of Cercidiphyllaceae (100.5 Ma), Loropetaleae (83.6 Ma), and Hamamelideae (83.6 Ma), respectively. These fossils are either infructescences/fruits or well-preserved flowers with pollen [60, 61, 66], and their systematic positions have been robustly verified and extensively used for calibrating the phylogeny of angiosperms [e.g., 29, 62, 105]. We used the ML tree as a topological constraint. The uncorrelated log-normal relaxed clock model was employed, with a Yule tree prior and under a GTR sequence substitution model as recommended by Paup v4.0 [106]. The MCMC was run for 500 million generations, sampling a tree every 1000 generations, with the first 20% of trees being discarded as burn-in. The convergence of the MCMC stimulations was inspected using Tracer v1.7.1 [99]. The ESS of the estimated parameters was, in most cases, > 200, and it was always > 125. Files containing the sampled trees were combined and annotated using LogCombiner v1.10.4 and Treeannotator v1.10.4, respectively, using the BEAST package. The maximum clade credibility tree with median ages and 95% highest posterior density intervals for all nodes was visualized using Figtree v1.3.1 (http://tree.bio.ed.ac.uk/software/figtree/).

### Electronic supplementary material

Below is the link to the electronic supplementary material.

Supplementary Material 1

Supplementary Material 2

Supplementary Material 3

Supplementary Material 4

Supplementary Material 5

Supplementary Material 6

Supplementary Material 7

Supplementary Material 8

Supplementary Material 9

Supplementary Material 10

## Data Availability

The complete plastomes generated in this study are available at the NCBI GenBank database, with the accession numbers being presented in Table S1.

## Abbreviations

BP: Base pair
BS: Bootstrap
CTAB: Cetyltrimethylammonium bromide
DNA: Deoxyribonucleic acid
ESS: Effective Sample Size
GC: Guanine and Cytosine
GTR: Generalized Time Reversible
IR: Inverted repeat
ISC: Large single-copy
MCMC: Markov Chain Monte Carlo
ML: Maximum Likelihood
MPT: Most-parsimonious tree
PCG: Plastid protein-coding genes
PP: Posterior probability
rRNA: Ribosomal RNA
tRNA: Transfer RNA
SSC: Small single copy
TBR: Bisection-reconnection
WCS: The ?woody clade? in Saxifragales
