# Supplementary material for: Plastid phylogenomics and fossil evidence provide new insights into the evolutionary complexity of the ‘woody clade’ in Saxifragales
Source: BMC Plant Biol. 2024 Apr 12;24:277. doi: 10.1186/s12870-024-04917-9 (PMC11010409; doi:10.1186/s12870-024-04917-9)
Supplement: Supplementary file 1 — Supplementary Material 1 [file 12870_2024_4917_MOESM1_ESM.docx]

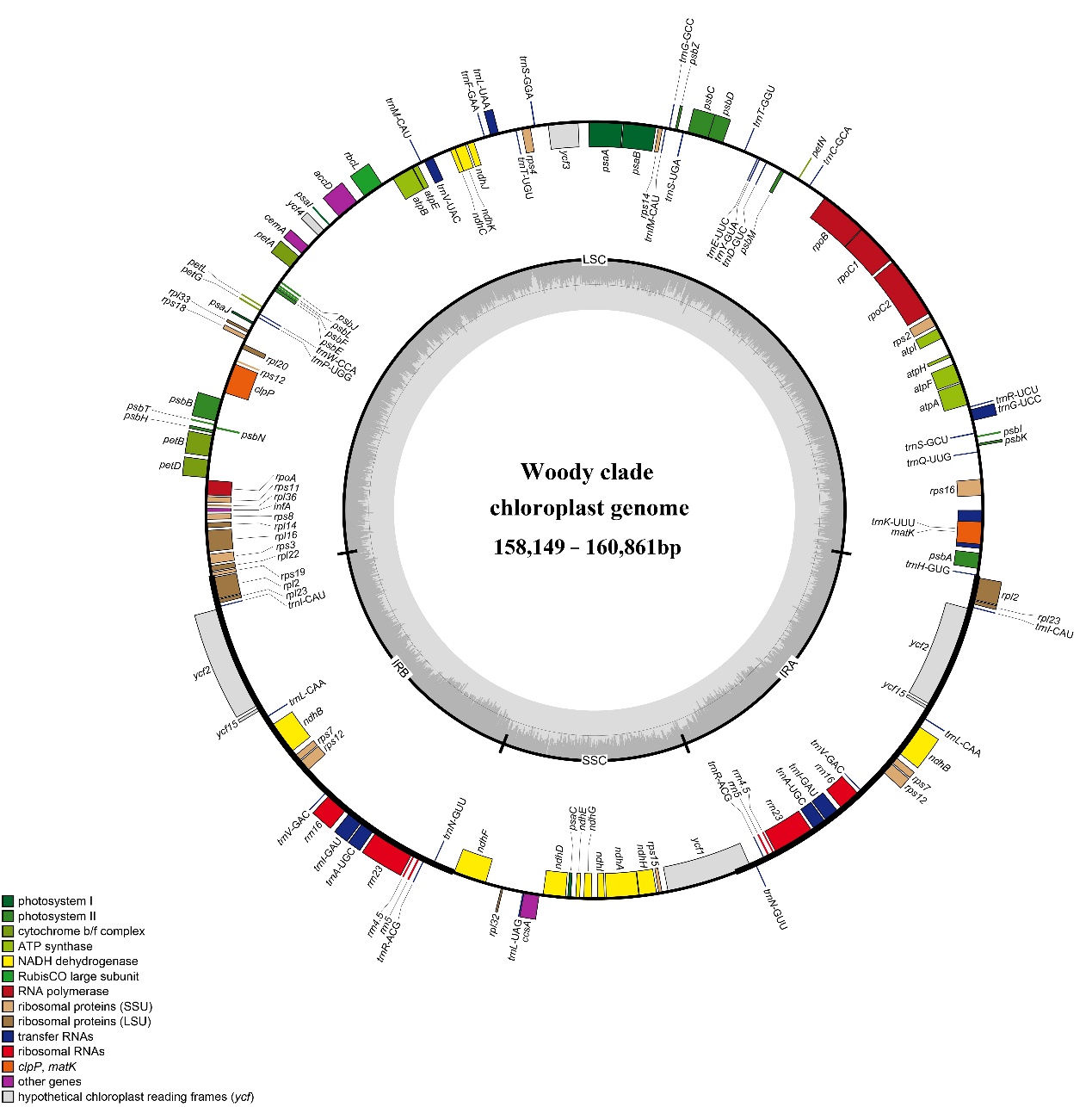


**Supplementary Material 1:** Fig. S1. Map of plastomes for the “woody clade” in Saxifragales. Genes shown outside the circle are transcribed clockwise, and those inside are transcribed counterclockwise. The colored bars indicate the known protein-coding genes, tRNA, and rRNA. The darker gray area of the inner circle denotes the GC content, while the lighter gray area indicates the AT content of the plastomes. LSC, large single-copy; SSC, small single-copy; IR, inverted repeat.
