# Supplementary material for: Plastid phylogenomics and fossil evidence provide new insights into the evolutionary complexity of the ‘woody clade’ in Saxifragales
Source: BMC Plant Biol. 2024 Apr 12;24:277. doi: 10.1186/s12870-024-04917-9 (PMC11010409; doi:10.1186/s12870-024-04917-9)
Supplement: Supplementary file 2 — Supplementary Material 2 [file 12870_2024_4917_MOESM2_ESM.docx]

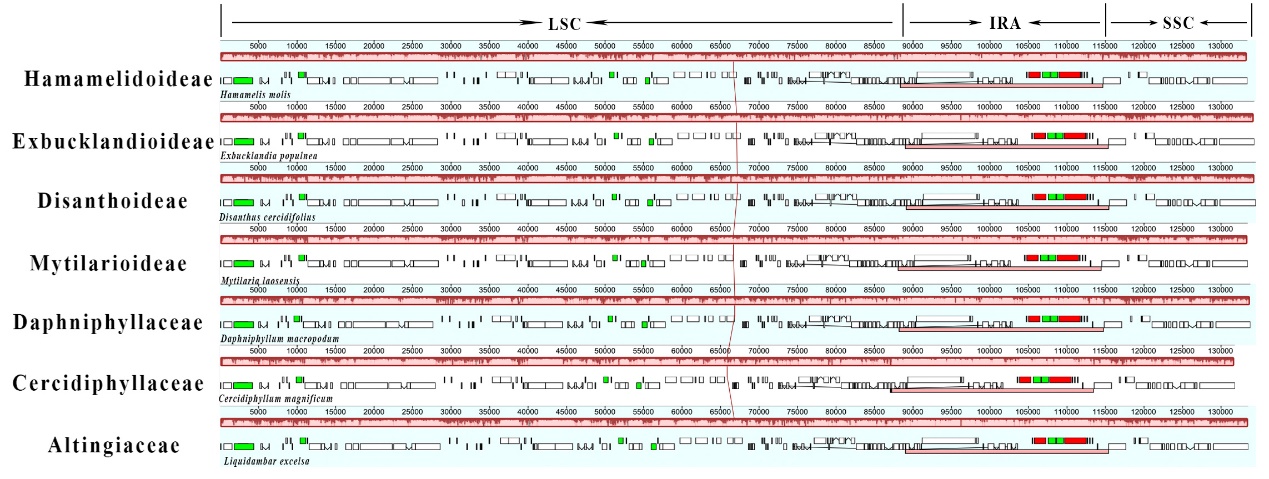


**Supplementary Material 2:** Fig. S2. Mauve alignment of plastomes for the “woody clade” in Saxifragales, with *Hamamelis mollis* (Hamamelidoideae) as the reference.
