# Supplementary material for: Plastid phylogenomics and fossil evidence provide new insights into the evolutionary complexity of the ‘woody clade’ in Saxifragales
Source: BMC Plant Biol. 2024 Apr 12;24:277. doi: 10.1186/s12870-024-04917-9 (PMC11010409; doi:10.1186/s12870-024-04917-9)
Supplement: Supplementary file 3 — Supplementary Material 3 [file 12870_2024_4917_MOESM3_ESM.docx]

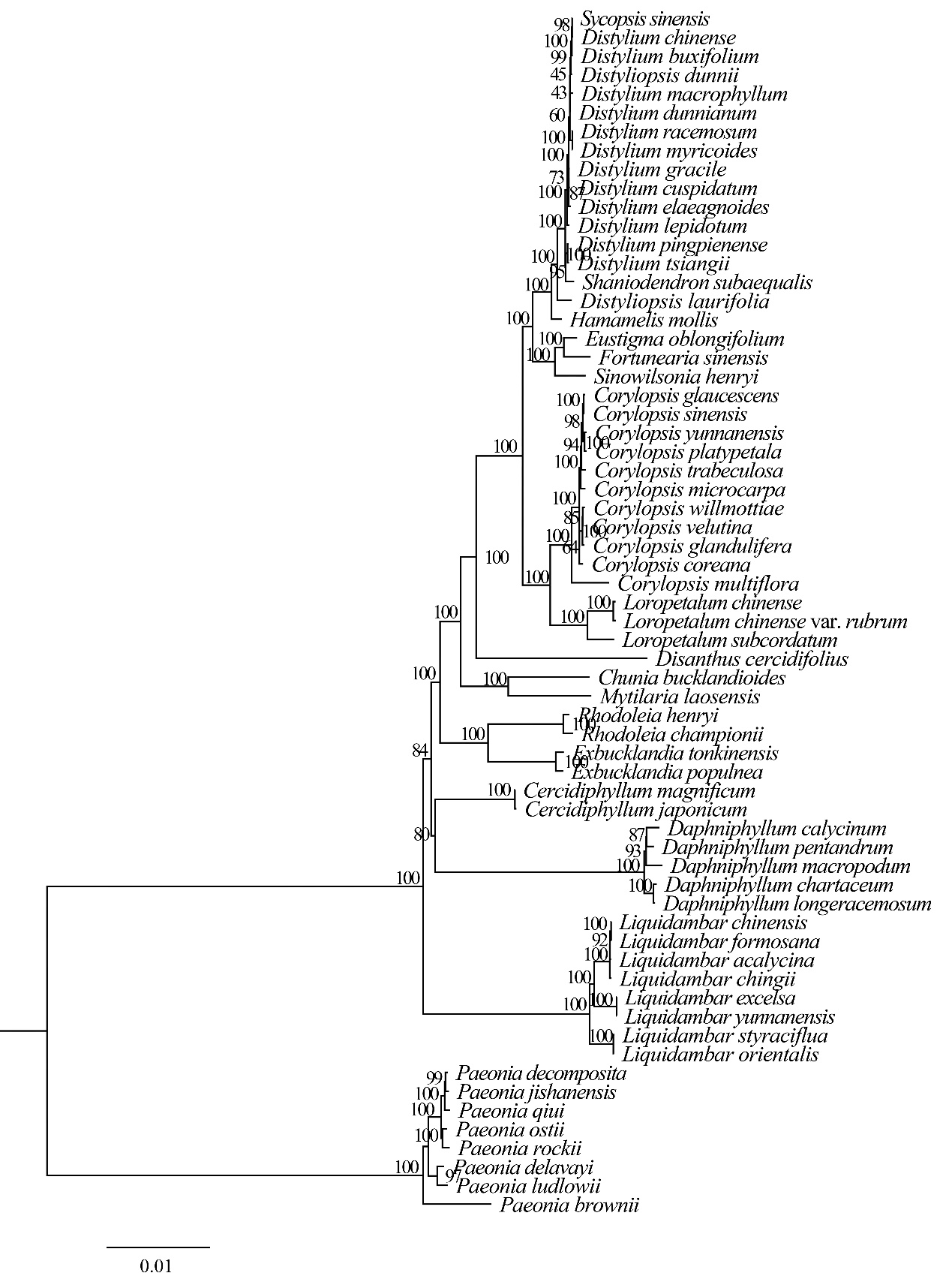


**Supplementary Material 3:** Fig. S3. Phylogenetic tree of the “woody clade” in Saxifragales based on maximum likelihood (ML) with 78 common protein coding genes of 64 species.
