# Supplementary material for: Plastid phylogenomics and fossil evidence provide new insights into the evolutionary complexity of the ‘woody clade’ in Saxifragales
Source: BMC Plant Biol. 2024 Apr 12;24:277. doi: 10.1186/s12870-024-04917-9 (PMC11010409; doi:10.1186/s12870-024-04917-9)
Supplement: Supplementary file 5 — Supplementary Material 5 [file 12870_2024_4917_MOESM5_ESM.docx]

Table S1 Taxa newly sequenced in this study with source, voucher, sequencing results, references for plastome assembly, and GenBank accession numbers.

| No. | Species | No. of total reads | No. of mapped reads | Coverage (×) | Reference | Voucher | Locality | GenBank accession |
| --- | --- | --- | --- | --- | --- | --- | --- | --- |
| 1 | *Corylopsis glaucescens* | 20,544,972 | 727,921 | 688.166 | MW043717 | Cai et al 14CS9766 | Gongshan, Yunnan, China | OP005511 |
| 2 | *Corylopsis platypetala* | 17,317,272 | 388,431 | 367.862 | MW043717 | WH-2012-0217 | Qiaojia, Yunnan, China | OP005515 |
| 3 | *Corylopsis sinensis* | 28,163,060 | 543,378 | 514.604 | MW043717 | Ji et Liu 2020035 | Cultivated in Kunming Botanical Garden, Yunnan, China | OP005507 |
| 4 | *Corylopsis trabeculosa* | 22,452,438 | 338,189 | 319.275 | MW043717 | Liu et al 14CS8523 | Gongshan, Yunnan, China | OP005529 |
| 5 | *Corylopsis willmottiae* | 12,550,166 | 268,420 | 254.206 | MW043717 | LiXJ089 | Emeishan, Sichuan, China | OP005517 |
| 6 | *Corylopsis yunnanensis* | 30,231,062 | 891,863 | 844.636 | MW043717 | Ji et Liu 2020036 | Cultivated in Kunming Botanical Garden, Yunnan, China | OP005523 |
| 7 | *Daphniphyllum calycinum* | 24,946,774 | 1,250,053 | 1183.859 | MH191390 | YangXF0336 | Shangsi, Guangxi, China | OP005513 |
| 8 | *Daphniphyllum chartaceum* | 17,104,472 | 495,660 | 469.413 | MH191390 | Guo et al 14CS9900 | Gongshan, Yunnan, China | OP005509 |
| 9 | *Daphniphyllum longeracemosum* | 30,838,978 | 1,035,678 | 980.836 | MH191390 | Ji et Liu 2020033 | Cultivated in Kunming Botanical Garden, Yunnan, China | OP005527 |
| 10 | *Distyliopsis dunnii* | 22,848,346 | 2,263,481 | 2143.623 | MN729500 | Yang et Zhao 3032 | Liancheng, Fujian, China | OP005522 |
| 11 | *Distyliopsis laurifolia* | 8,699,738 | 142,454 | 128.656 | MN729500 | Zhang et Liu 14CS8918 | Mile, Yunnan, China | OP005518 |
| 12 | *Distylium buxifolium* | 21,087,790 | 636,203 | 602.514 | MN729500 | GanQL750 | Zhuxi, Hubei, China | OP005516 |
| 13 | *Distylium dunnianum* | 19,631,876 | 212,537 | 201.283 | MN729500 | ZouFL0208 | Huaxi, Guizhou, China | OP005516 |
| 14 | *Distylium myricoides* | 9,868,786 | 451,873 | 424.481 | MN729500 | Ji et Liu 2020021 | Cultivated in Kunming Botanical Garden, Yunnan, China | OP005533 |
| 15 | *Distylium pingpienense* | 8,709,604 | 202,230 | 189.971 | MN729500 | Ji et Liu 2020022 | Cultivated in Kunming Botanical Garden, Yunnan, China | OP005519 |
| 16 | *Distylium racemosum* | 8,416,450 | 386,444 | 363.018 | MN729500 | Ji et Liu 2020020 | Cultivated in Kunming Botanical Garden, Yunnan, China | OP005514 |
| 17 | *Eustigma oblongifolium* | 13,844,130 | 172,547 | 163.41 | MG644608 | Zhang et al 17CS15579 | Longmen, Guangdong, China | OP005525 |
| 18 | *Exbucklandia populnea* | 7,895,906 | 464,592 | 436.429 | MW801175 | Ji et Liu 2020018 | Cultivated in Kunming Botanical Garden, Yunnan, China | OP005530 |
| 19 | *Exbucklandia tonkinensis* | 8,013,458 | 437,119 | 410.622 | MW801175 | Ji et Liu 2020017 | Cultivated in Kunming Botanical Garden, Yunnan, China | OP005520 |
| 20 | *Hamamelis mollis* | 8,652,626 | 661,798 | 621.681 | MH191387 | Ji et Liu 2020016 | Cultivated in Kunming Botanical Garden, Yunnan, China | OP005508 |
| 21 | *Liquidambar acalycina* | 9,176,748 | 762,310 | 716.1 | MT079213 | Ji et Liu 2020011 | Cultivated in Kunming Botanical Garden, Yunnan, China | OP005526 |
| 22 | *Liquidambar chinensis* | 22,257,424 | 1,946,171 | 1843.115 | MT079213 | LiangYL117 | Ganzhou, Jiangxi, China | OP005521 |
| 23 | *Liquidambar styraciflua* | 9,240,940 | 963,019 | 904.643 | MT079213 | Ji et Liu 2020012 | Cultivated in Kunming Botanical Garden, Yunnan, China | OP005532 |
| 24 | *Loropetalum chinense* | 8,508,296 | 58,387 | 54.848 | MZ642355 | Ji et Liu 2020009 | Cultivated in Kunming Botanical Garden, Yunnan, China | OP005528 |
| 25 | *Loropetalum chinense* var. rubrum | 7,363,066 | 316,635 | 297.441 | MZ642355 | Ji et Liu 2020010 | Cultivated in Kunming Botanical Garden, Yunnan, China | OP005531 |
| 26 | *Rhodoleia henryi* | 9,394,330 | 145,377 | 136.565 | MK834325 | Ji et Liu 2020014 | Cultivated in Kunming Botanical Garden, Yunnan, China | OP005510 |
| 27 | *Sycopsis sinensis* | 20,946,704 | 198,394 | 187.888 | MT323104 | Ya et Liu 15CS11178 | Huangshan, Anhui, China | OP005512 |
