# Supplementary material for: Plastid phylogenomics and fossil evidence provide new insights into the evolutionary complexity of the ‘woody clade’ in Saxifragales
Source: BMC Plant Biol. 2024 Apr 12;24:277. doi: 10.1186/s12870-024-04917-9 (PMC11010409; doi:10.1186/s12870-024-04917-9)
Supplement: Supplementary file 6 — Supplementary Material 6 [file 12870_2024_4917_MOESM6_ESM.docx]

Table S2 Plastome sequences downloaded from GenBank.

| No. | Family | Species | GenBank accesion |
| --- | --- | --- | --- |
| 1 | Altingiaceae | *Liquidambar excelsa* | MN106247 |
| 2 | Altingiaceae | *Liquidambar yunnanensis* | MN106248 |
| 3 | Altingiaceae | *Liquidambar cathayensis* | MN837678 |
| 4 | Altingiaceae | *Liquidambar formosana* | MT079213 |
| 5 | Altingiaceae | *Liquidambar orientalis* | MT079214 |
| 6 | Cercidiphyllaceae | *Cercidiphyllum magnificum* | MK550717 |
| 7 | Cercidiphyllaceae | *Cercidiphyllum japonicum* | MN496059 |
| 8 | Daphniphyllaceae | *Daphniphyllum oldhamii* | MH191390 |
| 9 | Daphniphyllaceae | *Daphniphyllum macropodum* | MN496060 |
| 10 | Hamamelidaceae | *Sinowilsonia henryi* | MF687003 |
| 11 | Hamamelidaceae | *Shaniodendron subaequalis* | MG334121 |
| 12 | Hamamelidaceae | *Loropetalum subcordatum* | MG457805 |
| 13 | Hamamelidaceae | *Chunia bucklandioides* | MG644608 |
| 14 | Hamamelidaceae | *Corylopsis coreana* | MG835449 |
| 15 | Hamamelidaceae | *Corylopsis glandulifera* | MZ642354 |
| 16 | Hamamelidaceae | *Corylopsis microcarpa* | MZ642356 |
| 17 | Hamamelidaceae | *Corylopsis multiflora* | MW043717 |
| 18 | Hamamelidaceae | *Corylopsis velutina* | MZ823391 |
| 19 | Hamamelidaceae | *Disanthus cercidifolius* | MK411769 |
| 20 | Hamamelidaceae | *Distylium chinense* | MW248112 |
| 21 | Hamamelidaceae | *Distylium cuspidatum* | MW248117 |
| 22 | Hamamelidaceae | *Distylium elaeagnoides* | MW248120 |
| 23 | Hamamelidaceae | *Distylium gracile* | MW248116 |
| 24 | Hamamelidaceae | *Distylium lepidotum* | MW248119 |
| 25 | Hamamelidaceae | *Distylium macrophyllum* | MN729500 |
| 26 | Hamamelidaceae | *Distylium tsiangii* | MN711651 |
| 27 | Hamamelidaceae | *Fortunearia sinensis* | MK533616 |
| 28 | Hamamelidaceae | *Mytilaria laosensis* | MN106252 |
| 29 | Hamamelidaceae | *Rhodoleia championii* | MK834325 |
| 30 | Paeoniaceae | *Paeonia delavayi* | KY817591 |
| 31 | Paeoniaceae | *Paeonia decomposita* | MG571273 |
| 32 | Paeoniaceae | *Paeonia brownii* | MH191385 |
| 33 | Paeoniaceae | *Paeonia jishanensis* | MK701988 |
| 34 | Paeoniaceae | *Paeonia ostii* | MK701990 |
| 35 | Paeoniaceae | *Paeonia qiui* | MT210544 |
| 36 | Paeoniaceae | *Paeonia rockii* | MW192444 |
| 37 | Paeoniaceae | *Paeonia ludlowii* | KY817592 |
