# Supplementary material for: Plastid phylogenomics and fossil evidence provide new insights into the evolutionary complexity of the ‘woody clade’ in Saxifragales
Source: BMC Plant Biol. 2024 Apr 12;24:277. doi: 10.1186/s12870-024-04917-9 (PMC11010409; doi:10.1186/s12870-024-04917-9)
Supplement: Supplementary file 7 — Supplementary Material 7 [file 12870_2024_4917_MOESM7_ESM.docx]

Table S3 Plastome features of the “woody clade” in Saxifragales.

| No. | Species | Total | | LSC | | SSC | | IR | | Coding sequences | | Uncoding sequences | |
| --- | --- | --- | --- | --- | --- | --- | --- | --- | --- | --- | --- | --- | --- |
|  |  | Size (bp) | GC (%) | Size (bp) | GC (%) | Size (bp) | GC (%) | Size (bp) | GC (%) | Size (bp) | GC (%) | Size (bp) | GC (%) |
| 1 | *Cercidiphyllum japonicum* | 159,897 | 37.9 | 88,056 | 36.0 | 18,973 | 32.4 | 26,434 | 43.0 | 91,194 | 40.4 | 68,703 | 34.6 |
| 2 | *Cercidiphyllum magnificum* | 159,899 | 37.9 | 88,058 | 36.0 | 18,973 | 32.4 | 26,434 | 43.0 | 91,401 | 40.4 | 68,498 | 34.6 |
| 3 | *Chunia bucklandioides* | 159,814 | 38.1 | 88,826 | 36.1 | 18,178 | 32.9 | 26,405 | 43.1 | 91,398 | 40.4 | 68,416 | 34.9 |
| 4 | *Corylopsis coreana* | 159,398 | 38.0 | 88,166 | 36.1 | 18,692 | 32.7 | 26,270 | 43.1 | 91,215 | 40.4 | 68,183 | 34.8 |
| 5 | *Corylopsis glaucescens* | 159,443 | 38.0 | 88,188 | 36.1 | 18,695 | 32.6 | 26,280 | 43.1 | 91,223 | 40.4 | 68,220 | 34.8 |
| 6 | *Corylopsis multiflora* | 158,993 | 38.0 | 87,895 | 36.1 | 18,672 | 32.6 | 26,213 | 43.1 | 91,172 | 40.4 | 67,821 | 34.8 |
| 7 | *Corylopsis platypetala* | 159,032 | 38.1 | 87,777 | 36.2 | 18,697 | 32.6 | 26,279 | 43.1 | 91,220 | 40.4 | 67,812 | 34.9 |
| 8 | *Corylopsis sinensis* | 159,454 | 38.0 | 88,190 | 36.1 | 18,706 | 32.7 | 26,279 | 43.1 | 91,217 | 40.4 | 68,237 | 34.8 |
| 9 | *Corylopsis trabeculosa* | 159,402 | 38.0 | 88,165 | 36.1 | 18,693 | 32.6 | 26,272 | 43.1 | 91,226 | 40.4 | 68,176 | 34.8 |
| 10 | *Corylopsis willmottiae* | 159,419 | 38.0 | 88,152 | 36.1 | 18,701 | 32.7 | 26,283 | 43.1 | 91,223 | 40.4 | 68,196 | 34.8 |
| 11 | *Corylopsis yunnanensis* | 159,441 | 38.0 | 88,189 | 36.1 | 18,694 | 32.7 | 26,279 | 43.1 | 91,223 | 40.4 | 68,218 | 34.8 |
| 12 | *Corylopsis glandulifera* | 159,402 | 38.0 | 88,134 | 36.1 | 18,702 | 32.6 | 26,283 | 43.1 | 91,986 | 40.3 | 67,416 | 34.9 |
| 13 | *Corylopsis microcarpa* | 159,438 | 38.0 | 88,185 | 36.1 | 18,693 | 32.6 | 26,280 | 43.1 | 91,518 | 40.4 | 67,920 | 34.8 |
| 14 | *Corylopsis velutina* | 159,414 | 38.0 | 88,146 | 36.1 | 18,702 | 32.7 | 26,283 | 43.1 | 91,242 | 40.4 | 68,172 | 34.9 |
| 15 | *Distylium chinense* | 159,087 | 38.0 | 87,830 | 36.2 | 18,791 | 32.5 | 26,233 | 43.1 | 92,075 | 40.3 | 67,012 | 34.8 |
| 16 | *Distylium cuspidatum* | 159,068 | 38.0 | 87,848 | 36.2 | 18,784 | 32.4 | 26,218 | 43.1 | 92,057 | 40.3 | 67,011 | 34.8 |
| 17 | *Distylium elaeagnoides* | 159,094 | 38.0 | 87,857 | 36.2 | 18,787 | 32.5 | 26,225 | 43.1 | 92,059 | 40.3 | 67,035 | 34.8 |
| 18 | *Distylium gracile* | 159,127 | 38.0 | 87,854 | 36.2 | 18,793 | 32.5 | 26,240 | 43.0 | 92,067 | 40.3 | 67,060 | 34.8 |
| 19 | *Distylium lepidotum* | 159,042 | 38.0 | 87,836 | 36.2 | 18,796 | 32.5 | 26,205 | 43.1 | 92,060 | 40.3 | 66,982 | 34.8 |
| 20 | *Distyliopsis dunnii* | 159,078 | 38.0 | 87,835 | 36.2 | 18,793 | 32.4 | 26,225 | 43.1 | 91,201 | 40.4 | 67,877 | 34.9 |
| 21 | *Distyliopsis laurifolia* | 159,328 | 38.0 | 88,122 | 36.1 | 18,770 | 32.5 | 26,218 | 43.1 | 91,166 | 40.4 | 68,162 | 34.8 |
| 22 | *Distylium buxifolium* | 159,079 | 38.0 | 87,825 | 36.2 | 18,788 | 32.5 | 26,233 | 43.0 | 91,201 | 40.4 | 67,878 | 34.9 |
| 23 | *Distylium chinense* | 159,118 | 38.0 | 87,855 | 36.2 | 18,783 | 32.5 | 26,240 | 43.1 | 91,201 | 40.4 | 67,917 | 34.9 |
| 24 | *Distylium dunnianum* | 159,087 | 38.0 | 87,857 | 36.2 | 18,772 | 32.5 | 26,229 | 43.1 | 91,177 | 40.4 | 67,910 | 34.9 |
| 25 | *Distylium macrophyllum* | 159,089 | 38.0 | 87,822 | 36.2 | 18,797 | 32.5 | 26,235 | 43.1 | 91,241 | 40.3 | 67,848 | 34.9 |
| 26 | *Distylium myricoides* | 159,108 | 38.0 | 87,864 | 36.2 | 18,782 | 32.5 | 26,231 | 43.1 | 91,183 | 40.4 | 67,925 | 34.9 |
| 27 | *Distylium pingpienense* | 159,146 | 38.0 | 87,877 | 36.2 | 18,785 | 32.5 | 26,242 | 43.1 | 91,177 | 40.4 | 67,969 | 34.9 |
| 28 | *Distylium tsiangii* | 159,125 | 38.0 | 87,897 | 36.2 | 18,788 | 32.4 | 26,220 | 43.1 | 91,666 | 40.3 | 67,459 | 34.8 |
| 29 | *Eustigma oblongifolium* | 159,104 | 38.1 | 87,889 | 36.3 | 18,763 | 32.8 | 26,226 | 43.1 | 91,132 | 40.4 | 67,972 | 35.0 |
| 30 | *Exbucklandia populnea* | 160,744 | 38.1 | 88,989 | 36.2 | 18,911 | 32.5 | 26,422 | 43.2 | 91,364 | 40.5 | 69,380 | 34.9 |
| 31 | *Exbucklandia tonkinensis* | 160,814 | 38.1 | 89,060 | 36.2 | 18,896 | 32.5 | 26,429 | 43.2 | 91,358 | 40.5 | 69,456 | 34.9 |
| 32 | *Fortunearia sinensis* | 159,441 | 38.1 | 88,124 | 36.3 | 18,781 | 32.9 | 26,268 | 43.1 | 91,212 | 40.5 | 68,229 | 35.0 |
| 33 | *Hamamelis mollis* | 159,766 | 38.0 | 88,349 | 36.1 | 18,751 | 32.5 | 26,333 | 43.1 | 91,198 | 40.4 | 68,568 | 34.8 |
| 34 | *Loropetalum chinense* | 159,099 | 38.0 | 87,856 | 36.1 | 18,727 | 32.7 | 26,258 | 43.1 | 91,192 | 40.4 | 67,907 | 34.8 |
| 35 | *Loropetalum chinense* var. *rubrum* | 159,425 | 38.0 | 88,141 | 36.1 | 18,772 | 32.7 | 26,256 | 43.1 | 91,192 | 40.4 | 68,233 | 34.8 |
| 36 | *Loropetalum subcordatum* | 158,706 | 38.0 | 88,216 | 36.1 | 18,494 | 32.7 | 25,998 | 43.1 | 90,808 | 40.4 | 67,898 | 34.9 |
| 37 | *Mytilaria laosensis* | 159,941 | 37.9 | 89,016 | 35.9 | 18,127 | 32.8 | 26,399 | 43.1 | 91,085 | 40.3 | 68,856 | 34.7 |
| 38 | *Shaniodendron subaequalis* | 159,324 | 38.0 | 87,968 | 36.1 | 18,932 | 32.4 | 26,212 | 43.1 | 91,356 | 40.3 | 67,968 | 34.9 |
| 39 | *Rhodoleia championii* | 159,115 | 37.7 | 88,144 | 35.8 | 18,131 | 32.3 | 26,420 | 42.9 | 91,191 | 40.2 | 67,924 | 34.4 |
| 40 | *Rhodoleia henryi* | 159,047 | 37.8 | 88,130 | 35.8 | 18,145 | 32.2 | 26,386 | 43.0 | 91,365 | 40.2 | 67,682 | 34.5 |
| 41 | *Sinowilsonia henryi* | 158,741 | 38.2 | 87,507 | 36.4 | 18,768 | 32.8 | 26,233 | 43.1 | 91,145 | 40.4 | 67,596 | 35.1 |
| 42 | *Sycopsis sinensis* | 159,084 | 38.0 | 87,828 | 36.2 | 18,790 | 32.5 | 26,233 | 43.1 | 91,201 | 40.4 | 67,883 | 34.9 |
| 43 | *Daphniphyllum calycinum* | 160,100 | 37.9 | 88,093 | 36.1 | 18,957 | 32.0 | 26,525 | 43.0 | 90,564 | 40.5 | 69,536 | 34.5 |
| 44 | *Daphniphyllum chartaceum* | 159,745 | 37.9 | 87,769 | 36.1 | 18,982 | 32.0 | 26,497 | 43.0 | 90,483 | 40.5 | 69,262 | 34.6 |
| 45 | *Daphniphyllum longeracemosum* | 159,977 | 37.9 | 88,001 | 36.1 | 18,982 | 32.0 | 26,497 | 43.0 | 90,504 | 40.5 | 69,473 | 34.6 |
| 46 | *Daphniphyllum macropodum* | 160,408 | 37.8 | 88,190 | 36.0 | 19,008 | 32.0 | 26,605 | 42.9 | 91,299 | 40.3 | 69,109 | 34.5 |
| 47 | *Daphniphyllum pentandrum* | 160,137 | 37.9 | 88,075 | 36.1 | 18,970 | 32.1 | 26,546 | 43.0 | 90,594 | 40.4 | 69,543 | 34.6 |
| 48 | *Disanthus cercidifolius* subsp. *longipes* | 158,149 | 37.9 | 87,139 | 36.0 | 18,290 | 32.3 | 26,360 | 43.0 | 91,080 | 40.3 | 67,069 | 34.7 |
| 49 | *Liquidambar acalycina* | 160,410 | 37.9 | 88,945 | 36.1 | 18,917 | 32.4 | 26,274 | 43.1 | 91,265 | 40.5 | 69,145 | 34.6 |
| 50 | *Liquidambar chinensis* | 160,428 | 37.9 | 88,960 | 36.1 | 18,920 | 32.4 | 26,274 | 43.1 | 91,263 | 40.5 | 69,165 | 34.6 |
| 51 | *Liquidambar chingii* | 160,444 | 37.9 | 88,969 | 36.1 | 18,913 | 32.4 | 26,281 | 43.1 | 91,238 | 40.5 | 69,206 | 34.6 |
| 52 | *Liquidambar excelsa* | 160,861 | 37.9 | 89,126 | 36.0 | 19,011 | 32.2 | 26,362 | 43.0 | 91,632 | 40.4 | 69,229 | 34.6 |
| 53 | *Liquidambar formosana* | 160,426 | 37.9 | 88,958 | 36.1 | 18,920 | 32.4 | 26,274 | 43.1 | 91,265 | 40.5 | 69,161 | 34.6 |
| 54 | *Liquidambar orientalis* | 160,771 | 37.9 | 88,882 | 36.1 | 18,947 | 32.4 | 26,471 | 43.1 | 91,259 | 40.5 | 69,512 | 34.6 |
| 55 | *Liquidambar styraciflua* | 160,732 | 37.9 | 88,850 | 36.1 | 18,960 | 32.4 | 26,461 | 43.0 | 91,265 | 40.5 | 69,467 | 34.6 |
| 56 | *Liquidambar yunnanensis* | 160,860 | 37.9 | 89,125 | 36.0 | 19,011 | 32.2 | 26,362 | 43.1 | 91,626 | 40.4 | 69,234 | 34.6 |
