# Supplementary material for: Plastid phylogenomics and fossil evidence provide new insights into the evolutionary complexity of the ‘woody clade’ in Saxifragales
Source: BMC Plant Biol. 2024 Apr 12;24:277. doi: 10.1186/s12870-024-04917-9 (PMC11010409; doi:10.1186/s12870-024-04917-9)
Supplement: Supplementary file 8 — Supplementary Material 8 [file 12870_2024_4917_MOESM8_ESM.docx]

Table S4 Characteristics of 78 protein-encoding genes for the “woody clade” in Saxifragales.

| No. | Genes | Aligned length (bp) | No. of parsimony informative sites | Divergence of parsimony informative sites | No. of variable sites | Divergence of variable sites |
| --- | --- | --- | --- | --- | --- | --- |
|  |  |  |  |  |  |  |
| 1 | *accD* | 1,494 | 233 | 15.60% | 297 | 19.88% |
| 2 | *atpA* | 1,527 | 157 | 10.28% | 196 | 12.84% |
| 3 | *atpB* | 1,497 | 144 | 9.62% | 176 | 11.76% |
| 4 | *atpE* | 402 | 38 | 9.45% | 48 | 11.94% |
| 5 | *atpF* | 585 | 64 | 10.94% | 82 | 14.02% |
| 6 | *atpH* | 246 | 21 | 8.54% | 29 | 11.79% |
| 7 | *atpI* | 744 | 69 | 9.27% | 90 | 12.10% |
| 8 | *ccsA* | 990 | 179 | 18.08% | 230 | 23.23% |
| 9 | *cemA* | 690 | 83 | 12.03% | 111 | 16.09% |
| 10 | *clpP* | 591 | 95 | 16.07% | 116 | 19.63% |
| 11 | *matK* | 1,327 | 275 | 20.72% | 339 | 25.55% |
| 12 | *ndhA* | 1,095 | 143 | 13.06% | 175 | 15.98% |
| 13 | *ndhB* | 1,539 | 35 | 2.27% | 45 | 2.92% |
| 14 | *ndhC* | 363 | 30 | 8.26% | 38 | 10.47% |
| 15 | *ndhD* | 1,410 | 202 | 14.33% | 264 | 18.72% |
| 16 | *ndhE* | 303 | 32 | 10.56% | 44 | 14.52% |
| 17 | *ndhF* | 1,473 | 224 | 15.21% | 287 | 19.48% |
| 18 | *ndhG* | 531 | 69 | 12.99% | 95 | 17.89% |
| 19 | *ndhH* | 1,182 | 127 | 10.74% | 163 | 13.79% |
| 20 | *ndhI* | 480 | 54 | 11.25% | 70 | 14.58% |
| 21 | *ndhJ* | 477 | 35 | 7.34% | 58 | 12.16% |
| 22 | *ndhK* | 642 | 71 | 11.06% | 90 | 14.02% |
| 23 | *petA* | 966 | 120 | 12.42% | 148 | 15.32% |
| 24 | *petB* | 648 | 51 | 7.87% | 66 | 10.19% |
| 25 | *petD* | 495 | 46 | 9.29% | 53 | 10.71% |
| 26 | *petG* | 114 | 12 | 10.53% | 13 | 11.40% |
| 27 | *petL* | 96 | 8 | 8.33% | 13 | 13.54% |
| 28 | *petN* | 90 | 6 | 6.67% | 6 | 6.67% |
| 29 | *psaA* | 2,253 | 174 | 7.72% | 226 | 10.03% |
| 30 | *psaB* | 2,205 | 184 | 8.34% | 234 | 10.61% |
| 31 | *psaC* | 246 | 26 | 10.57% | 34 | 13.82% |
| 32 | *psaI* | 111 | 28 | 25.23% | 29 | 26.13% |
| 33 | *psaJ* | 135 | 14 | 10.37% | 17 | 12.59% |
| 34 | *psbA* | 1,062 | 70 | 6.59% | 106 | 9.98% |
| 35 | *psbB* | 1,527 | 143 | 9.36% | 172 | 11.26% |
| 36 | *psbC* | 1,386 | 102 | 7.36% | 130 | 9.38% |
| 37 | *psbD* | 1,062 | 81 | 7.63% | 98 | 9.23% |
| 38 | *psbE* | 252 | 14 | 5.56% | 20 | 7.94% |
| 39 | *psbF* | 120 | 3 | 2.50% | 8 | 6.67% |
| 40 | *psbH* | 222 | 31 | 13.96% | 36 | 16.22% |
| 41 | *psbI* | 111 | 5 | 4.50% | 7 | 6.31% |
| 42 | *psbJ* | 123 | 9 | 7.32% | 10 | 8.13% |
| 43 | *psbK* | 186 | 21 | 11.29% | 26 | 13.98% |
| 44 | *psbL* | 208 | 9 | 4.33% | 29 | 13.94% |
| 45 | *psbM* | 111 | 17 | 15.32% | 19 | 17.12% |
| 46 | *psbN* | 132 | 9 | 6.82% | 11 | 8.33% |
| 47 | *psbT* | 108 | 8 | 7.41% | 10 | 9.26% |
| 48 | *psbZ* | 189 | 21 | 11.11% | 27 | 14.29% |
| 49 | *rbcL* | 1,428 | 137 | 9.59% | 162 | 11.34% |
| 50 | *rpl2* | 369 | 34 | 9.21% | 35 | 9.49% |
| 51 | *rpl14* | 411 | 44 | 10.71% | 43 | 10.46% |
| 52 | *rpl16* | 831 | 25 | 3.01% | 55 | 6.62% |
| 53 | *rpl20* | 381 | 44 | 11.55% | 58 | 15.22% |
| 54 | *rpl22* | 353 | 94 | 26.63% | 114 | 32.29% |
| 55 | *rpl23* | 285 | 16 | 5.61% | 19 | 6.67% |
| 56 | *rpl33* | 201 | 30 | 14.93% | 41 | 20.40% |
| 57 | *rpl36* | 114 | 13 | 11.40% | 17 | 14.91% |
| 58 | *rpoA* | 873 | 138 | 15.81% | 174 | 19.93% |
| 59 | *rpoB* | 3,214 | 326 | 10.14% | 415 | 12.91% |
| 60 | *rpoC1* | 2,052 | 213 | 10.38% | 276 | 13.45% |
| 61 | *rpoC2* | 4,206 | 581 | 13.81% | 744 | 17.69% |
| 62 | *rps11* | 417 | 44 | 10.55% | 55 | 13.19% |
| 63 | *rps12* | 377 | 17 | 4.51% | 19 | 5.04% |
| 64 | *rps14* | 303 | 28 | 9.24% | 38 | 12.54% |
| 65 | *rps15* | 273 | 48 | 17.58% | 65 | 23.81% |
| 66 | *rps16* | 264 | 34 | 12.88% | 51 | 19.32% |
| 67 | *rps18* | 150 | 29 | 19.33% | 39 | 26.00% |
| 68 | *rps19* | 285 | 38 | 13.33% | 53 | 18.60% |
| 69 | *rps2* | 735 | 76 | 10.34% | 97 | 13.20% |
| 70 | *rps3* | 660 | 96 | 14.55% | 121 | 18.33% |
| 71 | *rps4* | 606 | 67 | 11.06% | 89 | 14.69% |
| 72 | *rps7* | 468 | 6 | 1.28% | 11 | 2.35% |
| 73 | *rps8* | 405 | 62 | 15.31% | 80 | 19.75% |
| 74 | *ycf1* | 6,036 | 1,344 | 22.27% | 1,671 | 27.68% |
| 75 | *ycf15* | 246 | 13 | 5.28% | 17 | 6.91% |
| 76 | *ycf2* | 6,951 | 299 | 4.30% | 395 | 5.68% |
| 77 | *ycf3* | 510 | 34 | 6.67% | 46 | 9.02% |
| 78 | *ycf4* | 555 | 79 | 14.23% | 91 | 16.40% |
|  | Total | 67,705 | 7,601 | 11.23% | 9,682 | 14.30% |
