# Supplementary material for: Plastid phylogenomics and fossil evidence provide new insights into the evolutionary complexity of the ‘woody clade’ in Saxifragales
Source: BMC Plant Biol. 2024 Apr 12;24:277. doi: 10.1186/s12870-024-04917-9 (PMC11010409; doi:10.1186/s12870-024-04917-9)
Supplement: Supplementary file 9 — Supplementary Material 9 [file 12870_2024_4917_MOESM9_ESM.docx]

Table S5 The best-fitting model for molecular evolution in each dataset partition (Partition Finder).

| Partition | Best model | Genes |
| --- | --- | --- |
| 1 | GTR+G | *accD* (1st pos)*, rps18* (2nd pos)*, rpl33* (2nd pos)*, petN* (3rd pos)*, rps18* (1st pos) |
| 2 | GTR+G | *rps8* (2nd pos)*, accD* (2nd pos)*, psbL* (1st pos) |
| 3 | GTR+G | *accD* (3rd pos)*, psbB* (3rd pos)*, psaI* (1st pos)*, atpH* (3rd pos)*, ndhE* (3rd pos) |
| 4 | GTR | *atpA* (1st pos) |
| 5 | GTR | *psbM* (2nd pos)*, atpA* (2nd pos)*, psbT* (1st pos)*, atpB* (2nd pos)*, ndhI* (2nd pos) |
| 6 | GTR+G | *rps3* (3rd pos)*, atpA* (3rd pos) |
| 7 | GTR+G | *atpB* (1st pos)*, rpl16* (1st pos)*, atpI* (1st pos) |
| 8 | GTR+G | *atpB* (3rd pos)*, ndhK* (3rd pos) |
| 9 | GTR+I+G | *atpE* (1st pos) |
| 10 | GTR+G | *rpl23* (3rd pos)*, rpl2* (3rd pos)*, atpE* (2nd pos)*, petA* (2nd pos) |
| 11 | GTR+G | *psaA* (3rd pos)*, atpE* (3rd pos)*, ndhC* (3rd pos) |
| 12 | GTR+I+G | *rps11* (1st pos)*, rps2* (1st pos)*, petA* (1st pos)*, atpF* (1st pos) |
| 13 | GTR+I+G | *psbJ* (3rd pos)*, rps14* (2nd pos)*, ndhG* (1st pos)*, ndhA* (1st pos)*, cemA* (1st pos)*, atpF* (2nd pos) |
| 14 | GTR+I+G | *rpl36* (3rd pos)*, ndhA* (3rd pos)*, atpF* (3rd pos) |
| 15 | GTR | *psbE* (2nd pos)*, psaC* (1st pos)*, psbE* (1st pos)*, psbD* (1st pos)*, atpH* (1st pos) |
| 16 | GTR+I+G | *psbI* (2nd pos)*, ndhC* (2nd pos)*, psbN* (2nd pos)*, atpH* (2nd pos)*, psaB* (2nd pos)*, psbC* (2nd pos)*, psbD* (2nd pos)*, rps7* (2nd pos)*, psaC* (2nd pos)*, rpl36* (2nd pos) |
| 17 | GTR+G | *atpI* (2nd pos)*, psbZ* (2nd pos)*, petG* (2nd pos) |
| 18 | GTR+G | *rpoC1* (3rd pos)*, atpI* (3rd pos)*, rpoC2* (3rd pos) |
| 19 | GTR+G | *rps15* (1st pos)*, ccsA* (1st pos)*, rps2* (3rd pos) |
| 20 | GTR+G | *ccsA* (2nd pos) |
| 21 | GTR+G | *ccsA* (3rd pos)*, rps15* (3rd pos)*, ndhD* (3rd pos) |
| 22 | GTR+I+G | *rps15* (2nd pos)*, rpl33* (1st pos)*, petL* (3rd pos)*, cemA* (2nd pos) |
| 23 | GTR+G | *cemA* (3rd pos) |
| 24 | GTR+I+G | *clpP* (1st pos) |
| 25 | GTR+I+G | *clpP* (2nd pos) |
| 26 | GTR+G | *rpl33* (3rd pos)*, clpP* (3rd pos) |
| 27 | GTR+G | *matK* (1st pos) |
| 28 | GTR+G | *ycf3* (3rd pos)*, rps4* (3rd pos)*, petG* (3rd pos)*, psbK* (3rd pos)*, matK* (2nd pos) |
| 29 | GTR+G | *matK* (3rd pos) |
| 30 | GTR+I+G | *ndhA* (2nd pos) |
| 31 | GTR+I+G | *rps7* (1st pos)*, ndhB* (1st pos)*, rpl2* (2nd pos)*, psbA* (1st pos)*, petN* (2nd pos)*, rps12* (1st pos)*, petN* (1st pos)*, psaB* (1st pos) |
| 32 | GTR+I+G | *ndhB* (2nd pos)*, rpl14* (2nd pos)*, psaA* (2nd pos)*, psbA* (2nd pos)*, psbJ* (2nd pos)*, psbF* (2nd pos) |
| 33 | GTR+I+G | *ndhE* (2nd pos)*, rpoC1* (2nd pos)*, rps19* (2nd pos)*, rpl23* (2nd pos)*, ndhB* (3rd pos)*, ndhJ* (2nd pos)*, rps14* (1st pos)*, ycf2* (2nd pos) |
| 34 | GTR+G | *ndhH* (1st pos)*, ndhC* (1st pos)*, ndhK* (1st pos) |
| 35 | GTR+G | *ndhD* (1st pos)*, ycf4* (1st pos)*, psbL* (2nd pos) |
| 36 | GTR+G | *ndhD* (2nd pos) |
| 37 | GTR+I+G | *ndhE* (1st pos)*, rps8* (1st pos)*, ycf15* (1st pos)*, petL* (1st pos)*, psbM* (1st pos) |
| 38 | GTR+I+G | *rpl20* (2nd pos)*, ndhF* (1st pos) |
| 39 | GTR+I+G | *psaI* (2nd pos)*, psaJ* (1st pos)*, psbK* (1st pos)*, ndhF* (2nd pos)*, ndhG* (2nd pos) |
| 40 | GTR+G | *ndhF* (3rd pos) |
| 41 | GTR+G | *ndhG* (3rd pos) |
| 42 | GTR | *ndhH* (2nd pos)*, rps7* (3rd pos) |
| 43 | GTR+G | *rbcL* (3rd pos)*, psaJ* (3rd pos)*, ndhH* (3rd pos) |
| 44 | GTR+G | *rpoC2* (2nd pos)*, ndhI* (1st pos) |
| 45 | GTR+G | *ndhI* (3rd pos)*, rps11* (3rd pos)*, rpoA* (3rd pos) |
| 46 | GTR+I+G | *ycf4* (2nd pos)*, ndhK* (2nd pos)*, psbN* (1st pos)*, rpoB* (2nd pos)*, ndhJ* (1st pos) |
| 47 | GTR+I+G | *ndhJ* (3rd pos)*, psbH* (3rd pos)*, rps14* (3rd pos) |
| 48 | GTR+G | *petA* (3rd pos) |
| 49 | GTR+I+G | *petB* (1st pos) |
| 50 | GTR+I+G | *petB* (2nd pos) |
| 51 | GTR+I+G | *psaI* (3rd pos)*, petB* (3rd pos) |
| 52 | GTR+I+G | *petD* (1st pos) |
| 53 | GTR+I+G | *petD* (2nd pos) |
| 54 | GTR+I+G | *petD* (3rd pos) |
| 55 | GTR+I+G | *psbB* (2nd pos)*, psbT* (2nd pos)*, psbI* (1st pos)*, petG* (1st pos) |
| 56 | GTR+G | *petL* (2nd pos) |
| 57 | GTR+G | *psbC* (1st pos)*, psbJ* (1st pos)*, psbF* (1st pos)*, psaA* (1st pos) |
| 58 | GTR+G | *psaB* (3rd pos) |
| 59 | GTR+G | *psaC* (3rd pos)*, rpl22* (3rd pos) |
| 60 | GTR+G | *ycf2* (3rd pos)*, psaJ* (2nd pos) |
| 61 | GTR+G | *psbA* (3rd pos) |
| 62 | GTR+I+G | *psbB* (1st pos)*, rps11* (2nd pos)*, ycf3* (1st pos) |
| 63 | GTR+G | *psbC* (3rd pos)*, rpl14* (3rd pos) |
| 64 | GTR | *psbT* (3rd pos)*, psbD* (3rd pos)*, psbZ* (3rd pos) |
| 65 | GTR | *psbE* (3rd pos)*, psbN* (3rd pos) |
| 66 | GTR+G | *rpoA* (1st pos)*, psbF* (3rd pos)*, rpoC2* (1st pos) |
| 67 | GTR | *psbH* (1st pos) |
| 68 | GTR+G | *psbH* (2nd pos)*, rps2* (2nd pos)*, psbZ* (1st pos) |
| 69 | GTR+I+G | *rpoB* (3rd pos)*, psbI* (3rd pos) |
| 70 | GTR | *psbK* (2nd pos) |
| 71 | GTR+G | *rps12* (2nd pos)*, rps4* (2nd pos)*, rps3* (2nd pos)*, psbL* (3rd pos) |
| 72 | GTR+G | *ycf1* (3rd pos)*, psbM* (3rd pos) |
| 73 | GTR+I+G | *rbcL* (1st pos) |
| 74 | GTR+I+G | *rbcL* (2nd pos) |
| 75 | GTR+G | *rpoC1* (1st pos)*, rpl14* (1st pos) |
| 76 | GTR+I+G | *rpl16* (2nd pos) |
| 77 | GTR+I+G | *rpl16* (3rd pos) |
| 78 | GTR+I+G | *rpl2* (1st pos) |
| 79 | GTR+G | *rpl20* (1st pos)*, rpoA* (2nd pos) |
| 80 | GTR+G | *rpl20* (3rd pos) |
| 81 | GTR+G | *rpl22* (1st pos) |
| 82 | GTR+G | *ycf4* (3rd pos)*, rpl22* (2nd pos)*, rps18* (3rd pos) |
| 83 | GTR+G | *rpl23* (1st pos)*, ycf15* (3rd pos)*, ycf2* (1st pos)*, ycf15* (2nd pos)*, rpl36* (1st pos) |
| 84 | GTR+G | *rpoB* (1st pos) |
| 85 | GTR+I+G | *rps12* (3rd pos) |
| 86 | GTR+I+G | *rps16* (1st pos) |
| 87 | GTR+G | *rps16* (2nd pos) |
| 88 | GTR+I+G | *rps16* (3rd pos) |
| 89 | GTR+G | *rps19* (1st pos)*, rps4* (1st pos) |
| 90 | GTR+G | *rps19* (3rd pos) |
| 91 | GTR+G | *rps3* (1st pos) |
| 92 | GTR+G | *rps8* (3rd pos) |
| 93 | GTR+I+G | *ycf1* (1st pos) |
| 94 | GTR+I+G | *ycf1* (2nd pos) |
| 95 | GTR | *ycf3* (2nd pos) |
