# Supplementary material for: Plastid phylogenomics and fossil evidence provide new insights into the evolutionary complexity of the ‘woody clade’ in Saxifragales
Source: BMC Plant Biol. 2024 Apr 12;24:277. doi: 10.1186/s12870-024-04917-9 (PMC11010409; doi:10.1186/s12870-024-04917-9)
Supplement: Supplementary file 10 — Supplementary Material 10 [file 12870_2024_4917_MOESM10_ESM.docx]

Table S6 The best-fitting model for molecular evolution in each dataset partition (IQTREE).

| Partition | Best model | Genes |
| --- | --- | --- |
| 1 | F81+F | *psbL* (1st pos), *atpA* (2nd pos), *rpl23* (1st pos), *rpl23* (3rd pos), *atpI* (2nd pos), *atpB* (2nd pos), *ycf15* (2nd pos), *psbK* (2nd pos), *ndhE* (2nd pos), *psaI* (2nd pos), *psbD* (1st pos), *ycf3* (2nd pos), *psbT* (2nd pos), *atpH* (1st pos), *ndhC* (2nd pos) |
| 2 | F81+F+I | *petA* (1st pos), *ndhG* (2nd pos), *psbC* (1st pos), *atpE* (1st pos), *psbD* (2nd pos) |
| 3 | F81+F+I+G4 | *rbcL* (1st pos) |
| 4 | F81+F+R2 | *rps12* (3rd pos) |
| 5 | GTR+F+R3 | *ndhF* (3rd pos) |
| 6 | HKY+F | *ndhD* (2nd pos), *atpH* (3rd pos), *rpl20* (1st pos), *atpA* (1st pos), *psbL* (3rd pos), *rps15* (2nd pos), *petG* (3rd pos), *rps19* (1st pos), *psbK* (3rd pos), *rpl33* (1st pos), *rps7* (3rd pos), *psbZ* (2nd pos) |
| 7 | HKY+F+I | *rpoA* (2nd pos), *atpB* (1st pos), *ndhA* (2nd pos), *rps11* (1st pos), *psbA* (1st pos), *rpl16* (1st pos), *psaA* (2nd pos), *ndhB* (2nd pos), *petL* (2nd pos) |
| 8 | HKY+F+I+G4 | *rpl2* (1st pos) |
| 9 | HKY+F+R2 | *ndhF* (2nd pos) |
| 10 | JC | *ndhJ* (1st pos), *rpl23* (2nd pos), *ycf15* (3rd pos), *rpl2* (2nd pos), *psbT* (3rd pos), *rps19* (2nd pos), *psbK* (1st pos), *rps14* (1st pos), *psaB* (2nd pos), *psbF* (3rd pos), *petL* (1st pos), *rps7* (1st pos), *psbJ* (1st pos), *psbF* (1st pos), *rpl14* (2nd pos), *rpl36* (1st pos), *psbT* (1st pos), *psbI* (1st pos), *psbJ* (2nd pos), *psbF* (2nd pos), *psbE* (2nd pos), *psbE* (1st pos), *petN* (1st pos), *rps7* (2nd pos), *psaC* (1st pos), *petG* (1st pos), *rpl36* (2nd pos), *atpH* (2nd pos), *psbN* (2nd pos), *psaC* (2nd pos), *petN* (2nd pos), *psbI* (2nd pos) |
| 11 | JC+I | *clpP* (2nd pos), *atpF* (1st pos), *ycf3* (1st pos), *psbA* (2nd pos), *rps11* (2nd pos), *petN* (3rd pos), *psbC* (2nd pos) |
| 12 | JC+I+G4 | *rbcL* (2nd pos) |
| 13 | K2P | *atpI* (1st pos), *rps4* (2nd pos), *psbL* (2nd pos), *rpl33* (2nd pos), *rps18* (1st pos), *psaJ* (3rd pos), *ndhJ* (2nd pos), *rps12* (1st pos), *atpE* (2nd pos), *psbH* (1st pos), *psbH* (2nd pos), *psbN* (3rd pos), *psbM* (1st pos), *psbZ* (1st pos), *ndhI* (2nd pos), *psbN* (1st pos) |
| 14 | K2P+G4 | *rpl22* (1st pos), *rpl22* (2nd pos) |
| 15 | K2P+I | *rpoB* (2nd pos), *cemA* (1st pos), *ndhG* (1st pos), *rps16* (1st pos), *ndhK* (2nd pos), *rps12* (2nd pos), *ycf4* (2nd pos), *psbB* (2nd pos), *rps14* (2nd pos), *psaJ* (1st pos), *psaJ* (2nd pos) |
| 16 | K2P+R2 | *rps8* (2nd pos), *psaI* (3rd pos) |
| 17 | K3P | *psaA* (1st pos), *rps15* (1st pos), *rpl33* (3rd pos), *rps18* (2nd pos), *psaI* (1st pos) |
| 18 | K3P+G4 | *ndhA* (1st pos) |
| 19 | K3P+I | *rpoC1* (1st pos), *ndhK* (1st pos), *ycf4* (1st pos), *psaB* (1st pos), *ycf15* (1st pos) |
| 20 | K3P+I+G4 | *petB* (1st pos) |
| 21 | K3P+R2 | *rps2* (2nd pos), *rpl20* (2nd pos) |
| 22 | K3Pu+F | *ycf2* (3rd pos), *matK* (3rd pos), *psbD* (3rd pos), *rpl22* (3rd pos), *rpoA* (1st pos), *rps8* (3rd pos), *ycf3* (3rd pos), *atpE* (3rd pos), *rpl2* (3rd pos), *rps2* (1st pos), *rps18* (3rd pos), *psbH* (3rd pos), *psbZ* (3rd pos), *rps8* (1st pos), *rpl36* (3rd pos), *rpl14* (1st pos), *psbJ* (3rd pos), *psbI* (3rd pos) |
| 23 | K3Pu+F+G4 | *rpoC2* (2nd pos), *psaA* (3rd pos), *accD* (3rd pos), *rpoB* (3rd pos), *matK* (2nd pos), *atpB* (3rd pos), *ndhH* (3rd pos), *rpoA* (3rd pos), *psbA* (3rd pos), *ndhF* (1st pos), *rps3* (3rd pos), *atpI* (3rd pos), *ndhG* (3rd pos), *ycf4* (3rd pos), *clpP* (3rd pos), *rps11* (3rd pos), *rpl14* (3rd pos) |
| 24 | K3Pu+F+I | *ycf2* (2nd pos), *ycf2* (1st pos), *accD* (1st pos), *ndhD* (1st pos), *ccsA* (2nd pos), *ndhJ* (3rd pos), *ndhB* (3rd pos), *psbB* (1st pos) |
| 25 | K3Pu+F+R2 | *rpoC2* (3rd pos), *ndhD* (3rd pos), *matK* (1st pos), *rbcL* (3rd pos), *cemA* (3rd pos), *ndhI* (3rd pos), *rps19* (3rd pos), *psaC* (3rd pos), *atpF* (2nd pos), *psbM* (3rd pos) |
| 26 | K3Pu+F+R3 | *atpA* (3rd pos), *petD* (3rd pos) |
| 27 | TIM+F | *rps3* (2nd pos) |
| 28 | TIM+F+G4 | *atpF* (3rd pos), *rpl20* (3rd pos) |
| 29 | TIM+F+R2 | *rpl16* (3rd pos) |
| 30 | TIMe | *rps4* (1st pos), *ndhE* (1st pos) |
| 31 | TN+F | *psbM* (2nd pos), *petG* (2nd pos) |
| 32 | TN+F+I | *ndhB* (1st pos), *petB* (2nd pos) |
| 33 | TNe | *ndhI* (1st pos) |
| 34 | TNe+G4 | *rps16* (2nd pos) |
| 35 | TNe+I+G4 | *petD* (1st pos), *rpl16* (2nd pos) |
| 36 | TPM2+F+G4 | *cemA* (2nd pos) |
| 37 | TPM2+F+I+G4 | *petD* (2nd pos) |
| 38 | TPM3+F | *ndhK* (3rd pos), *rps2* (3rd pos), *rps4* (3rd pos) |
| 39 | TPM3+F+G4 | *rpoB* (1st pos), *accD* (2nd pos) |
| 40 | TPM3+F+I | *rpoC1* (2nd pos), *petA* (2nd pos), *rps14* (3rd pos) |
| 41 | TPM3+F+R3 | *rps16* (3rd pos) |
| 42 | TPM3u+F | *ndhE* (3rd pos), *ndhH* (2nd pos), *petL* (3rd pos) |
| 43 | TPM3u+F+G4 | *rps3* (1st pos) |
| 44 | TVM+F | *ndhA* (3rd pos), *rps15* (3rd pos), *ndhC* (3rd pos) |
| 45 | TVM+F+G4 | *ycf1* (2nd pos), *psaB* (3rd pos), *rpoC1* (3rd pos), *ccsA* (3rd pos), *psbB* (3rd pos), *psbC* (3rd pos), *petA* (3rd pos), *ndhH* (1st pos) |
| 46 | TVM+F+I | *rpoC2* (1st pos) |
| 47 | TVM+F+R2 | *ccsA* (1st pos), *petB* (3rd pos), *clpP* (1st pos) |
| 48 | TVM+F+R3 | *ycf1* (1st pos), *ycf1* (3rd pos) |
| 49 | TVMe | *ndhC* (1st pos) |
